# The kinetic landscape and interplay of protein networks in cytokinesis

**DOI:** 10.1016/j.isci.2020.101917

**Published:** 2020-12-11

**Authors:** Hiroki Okada, Brittany MacTaggart, Yoshikazu Ohya, Erfei Bi

**Affiliations:** 1 Department of Cell and Developmental Biology, Perelman School of Medicine, University of Pennsylvania, Philadelphia, PA 19104-6058, USA; 2 Department of Integrated Biosciences, Graduate School of Frontier Sciences, University of Tokyo, Kashiwa, Chiba, 277-8562, Japan

**Keywords:** Biological Sciences, Molecular Biology, Cell Biology

## Abstract

Cytokinesis is executed by protein networks organized into functional modules. Individual proteins within each module have been characterized to various degrees. However, the collective behavior and interplay of the modules remain poorly understood. In this study, we conducted quantitative time-lapse imaging to analyze the accumulation kinetics of more than 20 proteins from different modules of cytokinesis in budding yeast. This analysis has led to a comprehensive picture of the kinetic landscape of cytokinesis, from actomyosin ring (AMR) assembly to cell separation. It revealed that the AMR undergoes biphasic constriction and that the switch between the constriction phases is likely triggered by AMR maturation and primary septum formation. This analysis also provided further insights into the functions of actin filaments and the transglutaminase-like protein Cyk3 in cytokinesis and, in addition, defined Kre6 as the likely enzyme that catalyzes β-1,6-glucan synthesis to drive cell wall maturation during cell growth and division.

## Highlights

• Cytokinesis is executed by protein modules each with a unique kinetic signature
• Actomyosin ring constricts in a biphasic manner that is elaborately regulated
• The transglutaminase-like domain in Cyk3 plays a dual role in cytokinesis
• Kre6 catalyzes β-1,6-glucan synthesis at the cell surface during growth and division

## Graphical Abstract

**Figure:**
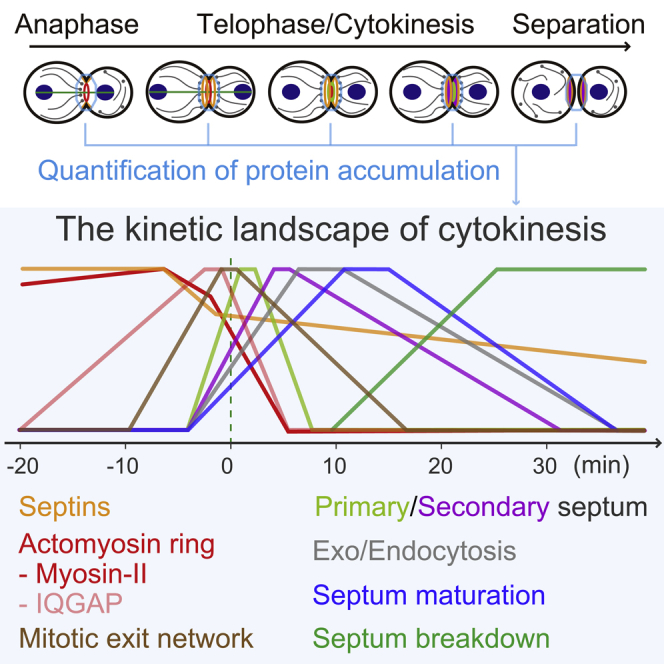

## Introduction

Cytokinesis, the last step of cell division, physically separates one cell into two cells. In fungal and animal cells, cytokinesis is accomplished by the spatiotemporally coordinated processes of a cortical actomyosin ring (AMR) constriction, targeted vesicle fusion, and localized extracellular matrix (ECM) remodeling (Bhavsar-Jog and Bi, 2017; Pollard, 2017; Willet, et al., 2015; Balasubramanian, et al., 2004). The AMR is thought to produce a contractile force that drives furrow ingression, as well as to guide targeted exocytosis that increases the surface area and delivers enzymatic cargoes for localized ECM remodeling at the division site (Wloka, et al., 2013; Fang, et al., 2010; Schmidt, et al., 2002; Vallen, et al., 2000). ECM remodeling is thought to stabilize the constricting AMR (Xu and Vogel, 2011; VerPlank and Li, 2005; Schmidt, et al., 2002; Bi, 2001). Thus, different elements of cytokinesis can regulate each other by a largely undefined mechanism to execute this fundamental process with high efficiency and fidelity.

Genome annotation indicates that more than 90 proteins localize to the bud neck, the cell division site of the budding yeast *Saccharomyces cerevisiae* (Handfield, et al., 2013; Huh, et al., 2003). Among these, ∼40 proteins have been identified to play a direct role in cytokinesis (Meitinger and Palani, 2016). These proteins can be assigned to at least five distinct modules: AMR, vesicle transport and exo-endocytosis, primary septum (PS) formation, secondary septum (SS) formation, and temporal coordination of cytokinetic events. Many of these proteins (described in Results) have been analyzed individually for their cellular behaviors and mutant phenotypes to define their roles in cytokinesis. However, what is common and unique about each component in a cytokinetic module and how different modules are temporally coordinated at the division site under normal and stressed conditions remain largely unknown.

Quantitative analysis of a multiprotein-driven process by imaging the molecular behaviors of the core components under the same experimental conditions has provided invaluable insights into the process. This approach has led to the establishment of the number and nanoscale architecture of proteins at the division site in fission yeast (McDonald, et al., 2017; Laplante, et al., 2016; Laporte, et al., 2011; Wu and Pollard, 2005) and of the temporal order and dynamic changes in local concentration and mobility of the cytokinetic proteins at the division site during the cell cycle in fission yeast and budding yeast, respectively (Wloka, et al., 2013; Wu, et al., 2006; Wu, et al., 2003). Microscopy-based kinetic analysis provides information on the dynamic accumulation of a protein at a given subcellular location, which can be very sensitive to environmental and genetic perturbations. This approach has proven powerful in understanding the fine control of timely SS formation by the F-BAR protein Hof1 (Oh, et al., 2017), the CDK regulation of IQGAP Iqg1 in AMR assembly (Naylor and Morgan, 2014), and vacuolar protein sorting in budding yeast (Casler and Glick, 2020). This approach has also provided mechanistic insights into the roles of the myosin-II isoforms Myo2 and Myp2 in cytokinesis under normal and stressed conditions in fission yeast (Okada, et al., 2019).

In this study, we used microscopy-based kinetic analysis to determine the accumulation kinetics of more than 20 core cytokinetic proteins from five different modules at the division site during the cell cycle at a 1-min time resolution. This analysis has provided a comprehensive temporal and kinetic picture of cytokinesis. More importantly, this analysis has revealed that proteins in each module share a kinetic signature, i.e., the peaking time, with some distinct attributes, whereas proteins from different modules display distinct signatures. At the mechanistic level, this analysis has also revealed that AMR constriction switches from a slow phase to a fast phase during furrow ingression, and this switch is controlled by the transglutaminase-like protein Cyk3-activated PS formation. Using the kinetic signature for functional prediction, coupled with validation experiments, we found that the type-II membrane protein Kre6 likely catalyzes β-1,6-glucan synthesis at the cell surface to drive cell wall maturation during cell growth and division.

## Results

### Strategy for quantitative and comparative analysis of protein accumulation kinetics at the division site during the cell cycle

**Figure 1:**
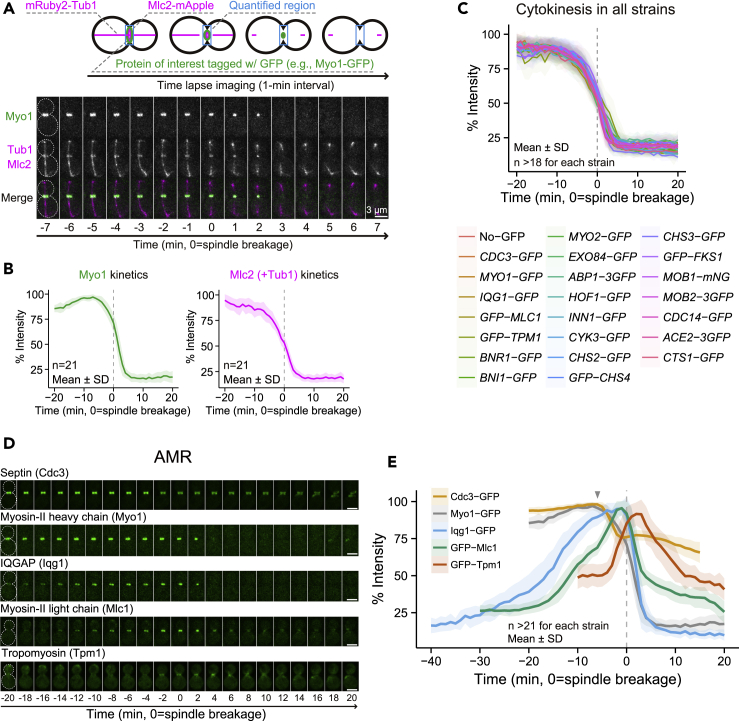
Quantification scheme of protein accumulation kinetics at the division site and the kinetics of proteins involved in AMR assembly (A) Schematic of quantification of cytokinetic proteins. Fluorescent intensity of GFP-tagged proteins (e.g., Myo1-GFP) at the division site (blue box) was measured from a series of time-lapse images. Representative images of the WT strain YEF9609 carrying Myo1-GFP that co-expressed with two cell cycle markers (mRuby2-Tub1 and Mlc2-mApple). Montages were created from selected frames of time-lapse series taken with a 1-min interval. The gray dotted line represents the cell outline. (B) Kinetics of Myo1-GFP and Mlc2-mApple (and sub-portion of mRuby2-Tub1). All data from individual cells were aligned with the timing of mitotic spindle breakage. See also Figure S1. (C) Kinetics of Mlc2-mApple of all analyzed strains. See also Figure S1. (D) Images of GFP-tagged proteins involved in AMR assembly. Montages of cells were created from selected frames of time-lapse series taken with a 1-min interval. The gray dotted line represents the cell outline. Strains used are as follows: YEF8434 (*CDC3-GFP mRuby2-TUB1 MLC2-mApple*), YEF9609 (*MYO1-GFP mRuby2-TUB1 MLC2-mApple*), YEF9610 (*IQG1-GFP mRuby2-TUB1 MLC2-mApple*), YEF8626 (*GFP-MLC1 mRuby2-TUB1 MLC2-mApple*), and YEF9583 (*GFP-TPM1 mRuby2-TUB1 MLC2-mApple*). All strains were cultured in SC medium. Scale bars, 3 μm. (E) Kinetics of the indicated GFP-tagged proteins in (D). Bold lines and associated shaded bands represent mean and SD values, respectively.

As a key step in understanding the assembly principles of the cytokinetic machinery, we performed quantitative live-cell imaging to compare the accumulation kinetics of more than 20 core cytokinetic proteins at the division site during cytokinesis (Figures 1A–1C). These proteins were carefully selected to represent different aspects of cytokinesis (Figure S1A). Unless noted otherwise, proteins were either N- or C-terminally tagged with GFP, indicated as GFP-X or X-GFP (X is the protein of interest), respectively. The tagged proteins were expressed from their endogenous loci controlled by their native promoters. To achieve an accurate comparison of the accumulation kinetics of each GFP-tagged protein from different strains, we included two RFP-based cell cycle markers as the “clock” in each strain (Figure 1A). mRuby2-Tub1, an RFP-tagged alpha-tubulin, marks the mitotic spindle (Markus, et al., 2015), and the spindle breakage is known to occur a few minutes before the onset of AMR constriction in wild-type (WT) cells (Pigula, et al., 2014; Woodruff, et al., 2010). Except where noted, time “0” refers to the point of spindle breakage or disassembly in all the analyses. Mlc2-mApple, an RFP-tagged regulatory light chain for the myosin-II heavy chain, Myo1, in budding yeast (Luo, et al., 2004), marks the AMR. Mlc2 co-localizes with Myo1 throughout the cell cycle, and its localization to the bud neck completely depends on its binding to Myo1 (Luo, et al., 2004). Importantly, the signal intensity and ring diameter of Myo1-GFP were highly correlated (Figure S1B), thus, the level of Myo1-GFP signal at the bud neck can be used to estimate the degree of AMR constriction or cytokinesis progression. As expected, the accumulation kinetics of Myo1-GFP and Mlc2-mApple were similar (Figures 1A and 1B), with the exception of a moderate decrease (−20 to −8 min) in Mlc2-mApple intensity that was presumably caused by the signal interference from a subset of mRuby2-Tub1 in the quantified region (Figures 1A and 1B). The kinetics of Mlc2-mApple, in relation to the spindle breakage, from all the strains imaged in this study were remarkably similar (Figures 1C and S1C), suggesting that our clock-based alignment of protein accumulation kinetics acquired from different strains is highly reliable. This result also indicates that none of the *GFP*-tagged genes significantly compromises cytokinesis progression.

### The actomyosin ring module: the switch of myosin constriction from the slow to the fast phase coincides with the completion of septin hourglass-to-double ring transition and the rapid arrival of tropomyosin at the division site

We first examined a set of proteins involved in AMR assembly and constriction. This includes septin Cdc3 (Caviston, et al., 2003), myosin-II heavy chain Myo1 (Bi, et al., 1998; Lippincott and Li, 1998), IQGAP Iqg1 (Lippincott and Li, 1998; Epp and Chant, 1997), the essential light chain for myosin-II Mlc1 (Feng, et al., 2015; Luo, et al., 2004; Boyne, et al., 2000; Shannon and Li, 2000; Stevens and Davis, 1998), and tropomyosin Tpm1 (Liu and Bretscher, 1992) (Figures 1D and 1E). Septins are required for the assembly of the AMR before cytokinesis (Shannon and Li, 2000; Bi, et al., 1998). Upon the mitotic exit, septins undergo the hourglass-to-double ring (HDR) transition that is accompanied by 20%–30% loss of signal intensity (McQuilken, et al., 2017; Wloka, et al., 2011). In accordance, the signal intensity of Cdc3-GFP (GFP inserted after residue 13 in Cdc3, Caviston, et al., 2003) decreased by 22.6% (−6 to −1 min) during this transition (Figures 1D and 1E). The onset of the HDR transition coincided with the onset of AMR constriction, as indicated by the initial drop in Myo1-GFP signal intensity (∼−6 min, arrowhead, Figures 1D, 1E, and S1B). Myo1-GFP exhibited a biphasic rate of constriction (Figure S1B), as seen for myosin-II during cytokinesis in fission yeast (Okada, et al., 2019), embryo cleavage in *Drosophila* (Xue and Sokac, 2016), or furrow ingression in mammalian cells (Wang, et al., 2019). The rate of constriction was initially slow (0.02 ± 0.02 μm/min, from −6 to −2 min), and then ramped up to ∼9-fold faster (0.18 ± 0.05 μm/min, from −2 to +4 min, Figure S1B). The slow and fast phases contributed 9.6% and 90.4% of the ring constriction (i.e., 0.10 ± 0.07 and 0.92 ± 0.11 μm in diameter), respectively (Figure S1B). Strikingly, the fast phase began after the completion of HDR transition, which supports the idea that septin clearance between the AMR and the plasma membrane (PM) during the HDR transition is required for AMR constriction (Chen, et al., 2020; Tamborrini, et al., 2018).

The yeast IQGAP Iqg1 is required for actin ring assembly (Lippincott and Li, 1998; Epp and Chant, 1997), Myo1 maintenance at the division site during cytokinesis (Fang, et al., 2010), and PS formation (Ko, et al., 2007; Korinek, et al., 2000). The accumulation of Iqg1-GFP at the bud neck began during G2/M (−40 min), rapidly increased during anaphase (from −15 min), peaked during AMR constriction (−2 min), and then disappeared quickly at the end of AMR constriction (Figures 1D and 1E). This pattern of localization is consistent with previous immunostaining results (Lippincott and Li, 1998; Epp and Chant, 1997). Because Mlc1, the essential light chain for Myo1 (Luo, et al., 2004), also binds to the IQ motifs of Myo2 (a myosin-V in yeast) and Iqg1 (Boyne, et al., 2000; Shannon and Li, 2000; Stevens and Davis, 1998) and is required for the localization of Iqg1 to the bud neck (Boyne, et al., 2000; Shannon and Li, 2000), we also analyzed its accumulation kinetics at the bud neck. Mlc1 was previously shown to slightly precede Iqg1 in localization to the bud neck by immunostaining (Boyne, et al., 2000; Shannon and Li, 2000). However, in our study, the accumulation of GFP-Mlc1 at the bud neck started during anaphase (−20 min), later than expected. Other parameters (the peak and disappearance) were similar to those of Iqg1 (Figures 1D and 1E). The delay in its initial recruitment could be due to (1) its initial localization being too faint and/or transient to be detected in live cells and/or (2) its localization being out-competed by the endogenous Mlc1, as the strain carried both *GFP-MLC1* and *MLC1* copies (Feng, et al., 2015).

Strikingly, the peaks of both Iqg1-GFP and GFP-Mlc1 coincided with the onset of the fast-phase Myo1-GFP constriction (−2 min). We hypothesized that both proteins could trigger the fast phase by promoting the assembly of the cytokinetic machinery, including the AMR and perhaps targeted vesicle fusion (see the section below). To examine this possibility further, we constructed GFP-Tpm1, a GFP-tagged yeast tropomyosin expressed by the *HIS3* promoter, to monitor the actin filaments at the division site. Tropomyosin-stabilized actin filaments are known to form the actin ring to mediate AMR constriction (Tolliday, et al., 2002), as well as the actin cables to guide exocytosis toward the division site during cytokinesis (Evangelista, et al., 2002; Sagot, et al., 2002). In support of our hypothesis, we found that GFP-Tpm1 began to localize to the division site as Iqg1 reached its peak (−3 min, Figures 1D and 1E). GFP-Tpm1 signal lingered after constriction (up to +12 min), which presumably reflects the actin cable-mediated exocytosis toward the division site.

### The vesicle transport and exo-endocytosis module: sequential assembly of the transport machine and spatiotemporal coupling of exo-endocytosis at the division site

**Figure 2:**
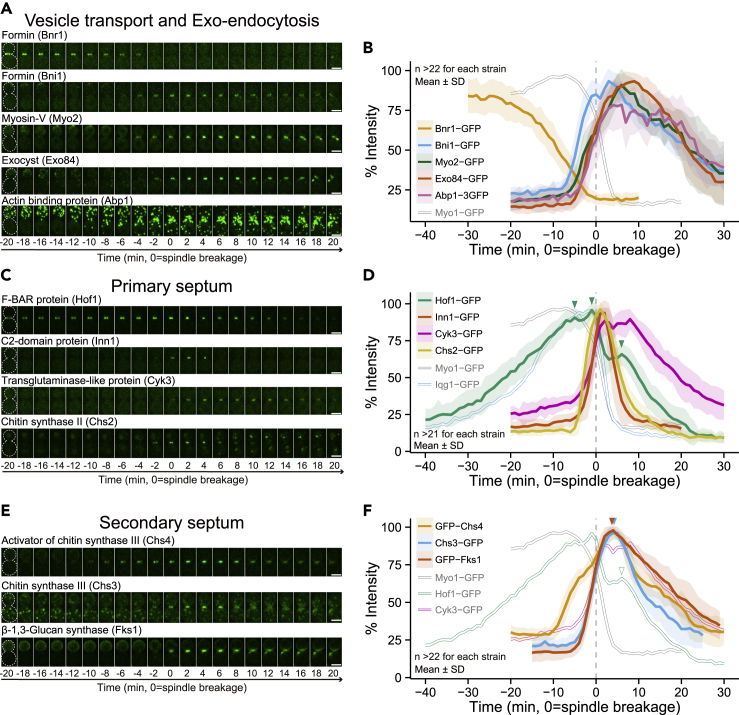
The kinetics of proteins involved in vesicle transport, PS formation, and SS formation (A, C, and E) Images of GFP-tagged proteins involved in (A) vesicle transport, (C) PS formation, and (E) SS formation. Montages of cells were created from selected frames of time-lapse series taken with a 1-min interval. The gray dotted line represents the cell outline. Strains used are as follows: (A) YEF9200 (*BNR1-GFP mRuby2-TUB1 MLC2-mApple*), YEF8535 (*BNI1-GFP mRuby2-TUB1 MLC2-mApple*), YEF8428 (*MYO2-GFP mRuby2-TUB1 MLC2-mApple*), YEF8432 (*EXO84-GFP mRuby2-TUB1 MLC2-mApple*), and YEF9198 (*ABP1-3GFP mRuby2-TUB1 MLC2-mApple*), (C) YEF8627 (*HOF1-GFP mRuby2-TUB1 MLC2-mApple*), YEF8628 (*INN1-GFP mRuby2-TUB1 MLC2-mApple*), YEF9197 (*CYK3-GFP mRuby2-TUB1 MLC2-mApple*), and YEF9611 (*CHS2-GFP mRuby2-TUB1 MLC2-mApple*), and (E) YEF9189 (*GFP-CHS4 mRuby2-TUB1 MLC2-mApple*), YEF9612 (*CHS3-GFP mRuby2-TUB1 MLC2-mApple*), and YEF8435 (*GFP-FKS1 mRuby2-TUB1 MLC2-mApple*). All strains were cultured in SC medium. Scale bars, 3 μm. (B, D, and F) Kinetics of the indicated GFP-tagged proteins in (A, C, and E), respectively. Bold lines and associated shaded bands represent mean and SD values, respectively. See also Figure S2.

The function of this module is to transport the post-Golgi vesicles to the division site for exocytosis to increase surface area and to deliver “enzymatic cargoes” (chitin synthases, glucan synthases, endochitinase, and glucanases) for localized ECM remodeling, i.e., cell wall synthesis and breakdown, at the division site (Bi and Park, 2012; Weiss, 2012). This module also includes endocytosis, which removes the enzymatic cargoes from the division site in a timely manner to prevent premature AMR constriction and spindle breakage (Chin, et al., 2016). The representatives of this module include the formins Bni1 and Bnr1 (Evangelista, et al., 1997; Imamura, et al., 1997), the type-V myosin Myo2 (Govindan, et al., 1995; Johnston, et al., 1991), the exocyst subunit Exo84 (Guo, et al., 1999), and the endocytic protein Abp1 (Holtzman, et al., 1993). Formins nucleate actin filaments that serve as the tracks for myosin-V-driven vesicle transport (Evangelista, et al., 2002; Sagot, et al., 2002). Two yeast formins (Bnr1 and Bni1) are known to switch their localization at the division site before cytokinesis (Orii, et al., 2016; Bloom, et al., 2011). The “early” formin (Bnr1-GFP) began to decrease at the division site during anaphase (-25 min) and was entirely removed during AMR constriction (−2 min, Figures 2A and 2B). In contrast, the “late” formin (Bni1-GFP) arrived at the division site slightly before the onset of AMR constriction (−8 min, Figures 2A and 2B). Strikingly, the arrival of Bni1-GFP at the division site was closely followed by Myo2-GFP and Exo84-GFP in succession (Figures 2A and 2B), which presumably reflects their order in the assembly and function of the transport module. The endocytic marker Abp1-3GFP appeared to sandwich the exocytic marker Exo84-GFP, and their kinetics of accumulation at the division site were remarkably similar (Figures 2A and 2B). This is consistent with the idea of spatiotemporal coupling of exo-endocytosis as seen during polarity establishment in budding yeast (Jose, et al., 2013) as well as in diverse processes across model systems (Grebnev, et al., 2017; Johansen, et al., 2016; Gundelfinger, et al., 2003; Battey, et al., 1999).

### The primary septum module: the actomyosin ring-associated activation of Chs2 for primary septum formation likely drives the fast-phase constriction

In budding yeast, the head of the myosin-II heavy chain Myo1, including its motor domain and light chain-binding sites, is responsible for only ∼20%–30% of the rate of AMR constriction, and the rest is attributed to PS formation, which follows the AMR and drives furrow ingression (Fang, et al., 2010; Lord, et al., 2005). The PS module includes the F-BAR protein Hof1 (Moravcevic, et al., 2015; Nishihama, et al., 2009), the C2-domain protein Inn1 (Nishihama, et al., 2009; Sanchez-Diaz, et al., 2008), the transglutaminase-like domain-containing protein Cyk3 (Nishihama, et al., 2009; Korinek, et al., 2000), and the chitin synthase-II Chs2 (an enzymatic cargo) (Chuang and Schekman, 1996; Sburlati and Cabib, 1986) (Figures 2C and 2D). Once the Chs2 cargo is delivered to the division site, Hof1, Inn1, and Cyk3 interact with each other to maintain and activate Chs2 at the division site for PS formation (Wang, et al., 2018; Foltman, et al., 2016; Nishihama, et al., 2009). Hof1-GFP displayed dramatic three-peak kinetics (at −5, −1, and +6 min, Figures 2D and S2A, green arrowheads). The first peak was nearly concurrent with the onset of the HDR transition, suggesting its association with the remodeling of septin hourglass (Figures S2A and S2B) (Meitinger, et al., 2011). The second peak, similar to that of its binding partner Iqg1 (Naylor and Morgan, 2014; Tian, et al., 2014), occurred at the onset of the fast-phase AMR constriction. This peak coincided with the acute recruitment of Chs2-GFP, Inn1-GFP, and Cyk3-GFP to the AMR (Figures 2C and 2D). The transition of Hof1 from its first peak to its second peak is consistent with its cell cycle-triggered shift from the septin hourglass to the AMR (Meitinger, et al., 2011). Together, these data suggest that the AMR-associated activation of Chs2 for PS formation (via the Iqg1-Hof1-Inn1-Cyk3-Chs2 complex) might drive the fast-phase AMR constriction. The third peak appeared to control the timely SS formation, as reported previously (Oh, et al., 2017). Thus, each discrete peak represents a distinct regulation and/or function of Hof1. Both Inn1 and Cyk3 are required for the activation of Chs2 (Devrekanli, et al., 2012; Nishihama, et al., 2009). Not surprisingly, they arrived at the division site at almost the same time as Chs2-GFP (Figures 2C and 2D). The kinetics of Inn1-GFP were nearly identical to those of Chs2-GFP, whereas the rates of the rise and fall of Cyk3-GFP were attenuated (Figures 2C and 2D). The slower rise of Cyk3-GFP is consistent with the observation that Hof1-Inn1 complex was readily detectable before AMR constriction, whereas Hof1-Cyk3 complex was detected only during cytokinesis (Wang, et al., 2018; Nishihama, et al., 2009). This also supports the hypothesis that the arrival of Cyk3 uncages the inhibitory effect of Chs2 by the C terminus of Inn1 at the division site (Foltman, et al., 2016). The fluctuating plateau (+2 to +8 min) followed by the slower fall of Cyk3-GFP is consistent with a role in SS formation (Onishi, et al., 2013). Together, these data suggest that the localization and activation of Chs2 at the division site for PS formation are temporally controlled with precision and are spatially coupled to the AMR to drive its fast-phase constriction and furrow ingression.

### The secondary septum module: Hof1 and Cyk3-based inhibitory mechanisms likely ensure the order of primary and secondary septum formation

At or near the end of PS formation, the SS that sandwiches the PS is synthesized by the chitin synthase-III (the catalytic subunit Chs3 and its activator Skt5/Chs4, hereafter Chs4) and the catalytic subunit of β-1,3-glucan synthase Fks1 (Oh, et al., 2017; Onishi, et al., 2013; Cabib, et al., 2001). As reported previously (Oh, et al., 2017), GFP-Chs4 preceded the arrival of Chs3-GFP at the division site, but both reached their peaks simultaneously (Figures 2E and 2F). This peak (∼+4 min, orange and blue arrowheads) was slightly after that of PS formation (+2 min, Figures 2C and 2D), in agreement with their order of formation. Hof1 competitively inhibits the binding of Chs4 to Chs3 to prevent precocious Chs3 activation during PS formation, and this inhibition is alleviated afterward to allow SS formation (Oh, et al., 2017). In line with this conclusion, Hof1-GFP suddenly dropped in intensity while Chs3-GFP and GFP-Chs4 were reaching their peaks (Figure 2F). Hof1 is also required for efficient removal of Chs4 after cytokinesis (Oh, et al., 2017). In line with this conclusion, the third peak of Hof1-GFP (+6 min, arrowhead) coincided with the rapid decrease of Chs4 at the division site (Figure 2F). Cyk3 inhibits Rho1-mediated activation of Fks1 for glucan synthesis during PS formation (Onishi, et al., 2013). Apparently, this inhibitory mechanism is attenuated during SS formation, as indicated by the small drop and then flattened peak of Cyk3-GFP while GFP-Fks1 was surging toward its peak (Figure 2F). Thus, the Hof1 and Cyk3-mediated inhibitory mechanisms ensure the timely activation of Chs3 and Fks1 for SS formation. The similar kinetics of Chs3-GFP and GFP-Fks1 (Figure 2F) suggest that chitin and glucan are concomitantly deposited in the SS. The removal of Fks1 and Cyk3 was slower than that of Chs3, suggesting that Cyk3-paced, Fks1-catalyzed glucan synthesis lasts longer than chitin synthesis during SS formation. Taken together, these data suggest that the timing of SS formation is dictated by the localization and activation of their synthetic enzymes at the division site, and that the Hof1 and Cyk3-based inhibitory mechanisms likely ensure the order of PS and SS formation.

### The temporal coordination module: the MEN and RAM ensure the order of furrow ingression and cell separation

**Figure 3:**
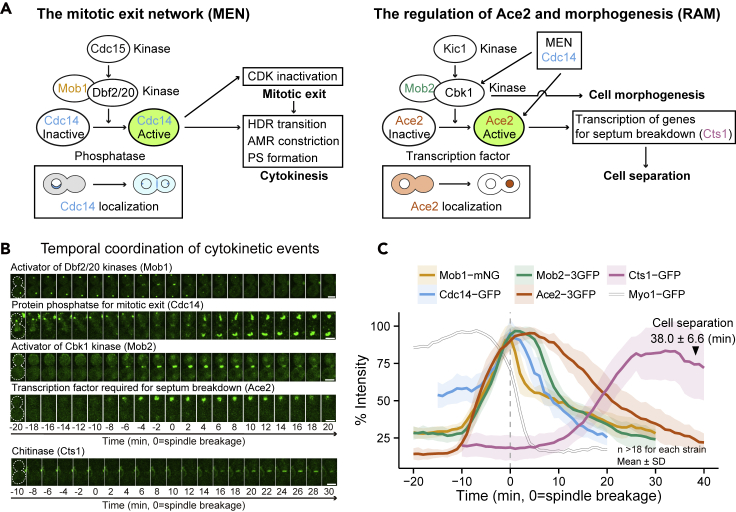
The kinetics of proteins involved in temporal coordination of cytokinetic events (A) Diagram of the signaling networks MEN and RAM in *S. cerevisiae*. The MEN controls mitotic exit and cytokinesis, whereas the RAM controls cell separation. See text for details. (B) Images of GFP-tagged proteins involved in the temporal control of cytokinesis and cell separation. Montages of cells were created from selected frames of time-lapse series taken with a 1-min interval. The gray dotted line represents the cell outline. Strains used are as follows: YEF8533 (*MOB1-mNG mRuby2-TUB1 MLC2-mApple*), YEF9819 (*CDC14-GFPmRuby2-TUB1 MLC2-mApple*), YEF8427 (*MOB2-3GFP mRuby2-TUB1 MLC2-mApple*), YEF9276 (*ACE2-3GFP mRuby2-TUB1 MLC2-mApple*), and YEF9186 (*CTS1-GFP mRuby2-TUB1 MLC2-mApple*). All strains were cultured in SC medium except for YEF9186, which was cultured in neutralized SC medium. Scale bars, 3 μm. (C) Kinetics of the indicated GFP-tagged proteins in (B). The intensity of Ace2-3GFP signal in the nucleus was measured. Bold lines and associated shaded bands represent mean and SD values, respectively. The timing of cell separation was scored from bright-field (BF) images.

To analyze how the cytokinetic events such as the AMR, PS and SS formation, and cell separation are temporally coordinated, we examined the mitotic exit network (MEN) and the regulation of Ace2 and morphogenesis (RAM) signaling pathways (Figure 3A) (Ma, et al., 2019; Weiss, 2012). The MEN controls mitotic exit by inactivating cyclin-dependent kinase (CDK) activity (Stegmeier and Amon, 2004) and controls cytokinesis by regulating the septin HDR transition, AMR constriction, and PS formation (Figure 3A) (Meitinger, et al., 2012; Oh, et al., 2012; Weiss, 2012). We performed time-lapse analysis of two MEN components, the upstream Mob1-mNeonGreen (mNG, the activator of the LATS-like kinases Dbf2 and Dbf20) and the downstream Cdc14-GFP (phosphatase) (Meitinger, et al., 2012; Weiss, 2012). In support of their roles in cytokinesis, the recruitment of the MEN components to the division site (-10 to -8 min) slightly preceded the onset of HDR transition and AMR constriction (-6 min), and both proteins reached their peaks during the fast-phase constriction (0 min, Figures 3B and 3C). Mob1-mNG disappeared more quickly than Cdc14-GFP, suggesting a more sustained functional requirement for Cdc14 at the division site.

The RAM controls cell morphogenesis and cell separation, the final step of cytokinesis (Figure 3A) (Weiss, 2012; Nelson, et al., 2003; Bidlingmaier, et al., 2001; Colman-Lerner, et al., 2001). The activation of RAM during cytokinesis was manifested by the upstream bud-neck localization of Mob2-3GFP (the activator of the Ndr-like kinase Cbk1) and the downstream daughter cell-specific nuclear localization of Ace2-3GFP (transcription factor, hence intensity was measured from the nucleus, Figure 3A) (Weiss, 2012; Nelson, et al., 2003; Bidlingmaier, et al., 2001; Colman-Lerner, et al., 2001). Both proteins arrived at their respective cellular locations at the same time as the MEN components but reached their peaks at later time points (Figures 3B and 3C). Similar to the MEN components, Ace2-3GFP disappeared from its functional location later than Mob2-3GFP. This is consistent with their order of actions and suggests a sustained requirement for Ace2 function. Importantly, Ace2-3GFP, which drives the expression of the endochitinase (Cts1-GFP) that degrades the PS during cell separation, reached its peak (+5 min) coincident with the initial recruitment of Cts1 to the division site. Cts1 accumulated there progressively until its peak (+25 min), which was followed by cell separation (+38 ± 6.6 min).

### Kinetic analysis of molecular response to actin filament disruption reveals mechanistic insights into the interplays between cytokinetic modules

**Figure 4:**
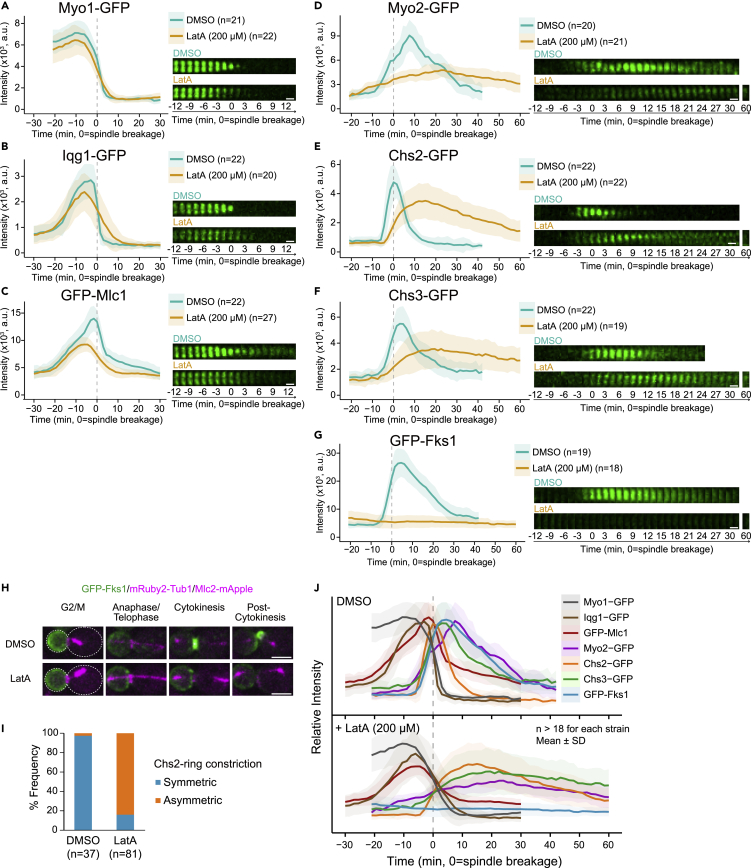
Effects of disrupting actin filaments on the kinetics of proteins involved in AMR assembly and septum formation (A–G) The impact of disrupting actin filaments on AMR constriction and septum formation. Effects of actin filament disruption by LatA (200 μM) on AMR constriction, exocytosis, and septum formation were determined by imaging a mixed culture of two strains YEF9198 and (A) YEF9609, (B) YEF9610, (C) YEF8626, (D) YEF8428, (E) YEF9611, (F) YEF9612, and (G) YEF8435. Kinetics were plotted as bold lines and associated shaded bands, which represent mean and SD values, respectively. Montages of division sites were created from selected frames of time-lapse series taken with a 1.5-min interval. See also Figure S3. Scale bars, 1 μm. (H) Montages of representative cells at the indicated cell cycle stages were created from selected frames of the time-lapse series acquired in (G). Scale bars, 3 μm. (I) Symmetry of Chs2 ring constriction was scored from the time-lapse data acquired in (E). (J) Summary of the kinetics of indicated cytokinetic proteins in the presence of DMSO or LatA. All kinetics obtained in (A–G) were superimposed in the two separate plots marked as DMSO or LatA.

The kinetic analyses described above indicate that different modules are coordinately regulated to ensure a functional order during cytokinesis under normal growth conditions. However, it remains poorly understood how different modules respond to chemical or genetic perturbations at the system level. To address this question, we first examined the modular response to filamentous actin (F-actin) disruption by latrunculin A (LatA) (Ayscough, et al., 1997). To ensure the disruption of F-actin in all the cells analyzed, we mixed Abp1-3GFP cells with our test cells before time-lapse analysis. Most of the actin patches were quickly disassembled (∼5 min) after the addition of 200 μM LatA (Figure S3A), and all the kinetics were acquired after this time point. The fact that all the Mlc2-mApple kinetics from our test strains aligned so tightly ensures a fair comparison (Figure S3B). We first analyzed the behaviors of the AMR components, Myo1-GFP, Iqg1-GFP, and GFP-Mlc1, in the absence of F-actin. All three proteins failed to constrict, as previously observed for Myo1 (Bi, et al., 1998), and all disassembled with a linear kinetic profile (Figures 4A–4C). However, Myo1-GFP did not shift its peak and only exhibited a mild reduction at the division site when compared with the DMSO control (91% of the control value at the peak, Table S3). In contrast, both Iqg1-GFP and GFP-Mlc1 left-shifted their peaks toward Myo1-GFP by 3 min and displayed a significant reduction at the division site (84% and 66% of their control values at the peaks for Iqg1-GFP and GFP-Mlc1, respectively, Table S3). These data indicate that the surge of Iqg1-GFP and GFP-Mlc1 after the Myo1-GFP peak, which correlates with the switch of Myo1-GFP constriction from the slow-phase to fast-phase (Figure 1E), depends on actin filaments. Consistent with our previous observation (Feng, et al., 2015), the localization of GFP-Mlc1 at the division site showed the strongest dependency on F-actin during cytokinesis. This is understandable, as Mlc1 binds not only to Myo1 and Iqg1 but also to Myo2 and secretory vesicles whose targeting to the bud neck was severely compromised by F-actin disruption (Figure 4D). Collectively, these data indicate that LatA treatment reduces the accumulation of these cytokinetic proteins at the division site to various degrees, left-shifts the peaks of Iqg1-GFP and GFP-Mlc1 toward that of Myo1-GFP, and causes them to disassemble with similar kinetics.

Next, we examined the kinetics of vesicle transport and cargo proteins in the presence of LatA. Strikingly, Myo2-GFP (myosin-V) displayed a dramatic reduction in both the rate and the magnitude of accumulation at the division site (12% and 53% of their control values, respectively), with its peak right-shifted by 16.5 min (Figure 4D and Table S3). A small yet significant fraction of Myo2-GFP displayed F-actin-independent localization at the division site, suggesting that Myo2 can be captured from the cytoplasm and retained at the division site by some cytokinetic proteins. Both Chs2-GFP and Chs3-GFP accumulated at the division site with much slower rates (37% and 29% of their control rates, respectively) and substantially reduced peak levels (73% and 65% of their control values, respectively), with their peaks also right-shifted by 15 and 19.5 min, respectively (Figures 4E and 4F, and Table S3). In contrast, virtually no GFP-Fks1 was accumulated at the division site (Figure 4G). This difference could be explained by their distinct trafficking routes. Chs2 and Chs3 are transported from the ER-Golgi system and chitosomes, respectively, to the PM (Ziman, et al., 1998; Chuang and Schekman, 1996). In contrast, GFP-Fks1 appeared to require endocytosis-mediated recycling from the bud membrane to the bud-neck membrane (Figure 4H), which suggests that there is no *de novo* synthesis of Fks1 during cytokinesis. LatA abolishes actin patch-mediated internalization of cargoes during endocytosis (Chin, et al., 2016; Ayscough and Drubin, 1998). As a result, GFP-Fks1 lingered at the bud membrane (Figure 4H) and Chs2-GFP and Chs3-GFP delayed their endocytic removal from the division site (Figures 4E and 4F). Collectively, these data indicate that LatA treatment significantly reduces the levels of the cargo enzymes at the division site and right-shifts their peaks away from that of Myo1.

The AMR is known to guide PS formation, and a compromised AMR structure leads to misoriented PS formation (Fang, et al., 2010; Schmidt, et al., 2002; Vallen, et al., 2000). In agreement with this, in LatA-treated cells, Chs2-GFP constricted asymmetrically (Figures 4E, 4I, and S3C). Simultaneous imaging of Chs2-GFP and chitin deposition (calcofluor white [CW]) in *chs1Δ chs3Δ* cells (in which Chs2 is the only remaining chitin synthase) in the presence of LatA showed that Chs2-GFP movement (green arrowheads) was strictly followed by chitin deposition (blue arrowheads, Figure S3C, 22 of 23 cells showed such a phenotype). This result demonstrates that the activation of Chs2 for PS formation does not require F-actin or AMR constriction, and also provides the visual evidence that chitin deposition is the driving force for furrow ingression in the absence of AMR constriction.

In summary, despite the specific and meaningful differences between individual proteins, the major impact of LatA treatment is to shift the peaks of the AMR components to the left slightly and shift the peaks of the synthetic cargo enzymes to the right significantly (Figure 4J). This increased temporal separation between the peaks of the AMR and PS formation components explains why Myo1 disassembles without constriction in LatA-treated cells (Bi, et al., 1998), as Myo1 disassembly is not followed by concomitant PS formation. This observation also suggests that Myo1 disassembly per se is not sufficient to trigger furrow ingression. Collectively, these data indicate that the localizations of the AMR components are much less dependent on actin filaments than those of the cargo enzymes, and that actin filaments are required for the coordinated functions of the AMR, vesicle transport, and septum formation.

### Genetic perturbation coupled with kinetic analysis defines the dual role of Cyk3 in the activation of PS formation and inhibition of SS formation

**Figure 5:**
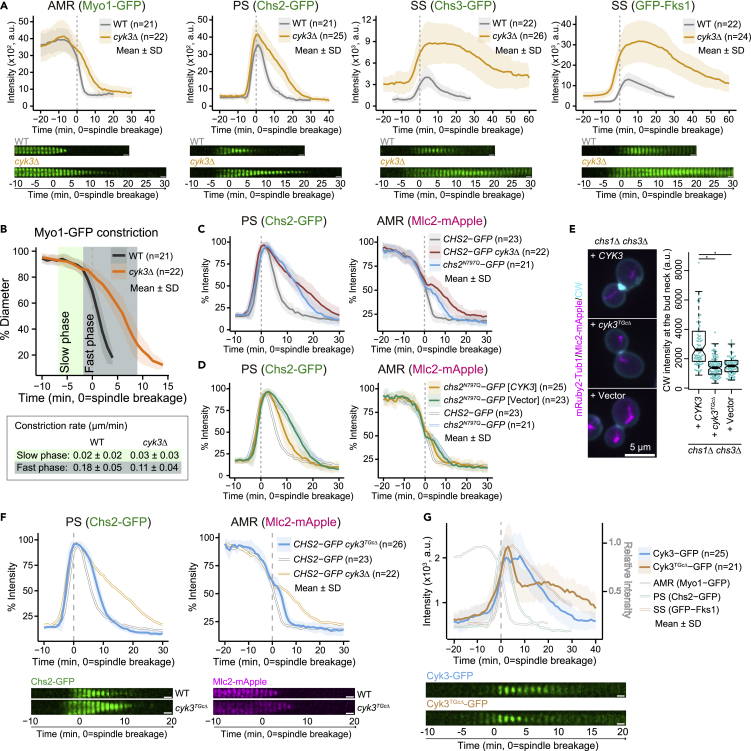
Functional analysis of Cyk3 and its TGc domain in fast-phase constriction and septum formation (A) Effects of loss of Cyk3 on the AMR behavior and septum formation were determined by imaging of WT and *cyk3*Δ strains: YEF9609, YEF9729 (*cyk3*Δ *MYO1-GFP mRuby2-TUB1 MLC2-mApple*), YEF9611, YEF9732 (*cyk3*Δ *CHS2-GFP mRuby2-TUB1 MLC2-mApple*), YEF9612, YEF9733 (*cyk3*Δ *CHS3-GFP mRuby2-TUB1 MLC2-mApple*), YEF8435, and YEF9242 (*cyk3*Δ *GFP-FKS1 mRuby2-TUB1 MLC2-mApple*). Kinetics were plotted as bold lines and associated shaded bands that represent mean and SD values, respectively. Montages of division sites were created from selected frames of time-lapse series taken with a 1-min interval. See also Figure S4. Scale bars, 1 μm. (B) Diameter of the Myo1 ring in WT and *cyk3*Δ cells during constriction. Data were acquired from the cells imaged in (A, left most images). Bold lines and associated shaded bands represent mean and SD values, respectively. (C) Effects of reduced Chs2 activity on septum formation (Chs2-GFP) and AMR constriction (Mlc2-mApple) were determined by imaging of WT, *cyk3*Δ, and *chs2*^*N797Q*^ strains: YEF10098 (*CHS2-GFP mRuby2-TUB1 MLC2-mApple*), YEF10153 (*cyk3*Δ *CHS2-GFP mRuby2-TUB1 MLC2-mApple*), and YEF10101 (*chs2*^*N797Q*^*-GFP mRuby2-TUB1 MLC2-mApple*). Kinetics were plotted as bold lines and associated shaded bands that represent mean and SD values, respectively. See also Figure S4. (D) Effects of overexpression of *CYK3* on septum formation (Chs2-GFP) and AMR constriction (Mlc2-mApple) were determined by imaging of *chs2*^*N797Q*^ strains carrying either *CYK3* high-copy plasmid or empty vector: YEF10128 (*chs2*^*N797Q*^*-GFP mRuby2-TUB1 MLC2-mApple* [2μ, *TRP1*, *CYK3*]) and YEF10129 (*chs2*^*N797Q*^*-GFP mRuby2-TUB1 MLC2-mApple* [2μ, *TRP1*]). The reference plots of *CHS2-GFP* and *chs2*^*N797Q*^*-GFP* were modified from (C). Kinetics were plotted as bold lines and associated shaded bands that represent mean and SD values, respectively. (E) The impact of the TGc domain of Cyk3 on PS formation (chitin stained by CW) was determined by imaging of *chs1Δ chs3Δ* strains carrying a high-copy plasmid containing *CYK3* or *cyk3*^*TGcΔ*^ or the empty vector alone: YEF10369 (*chs1Δ chs3Δ mRuby2-TUB1 MLC2-mApple* [2μ, *TRP1*, *CYK3*]), YEF10370 (*chs1Δ chs3Δ mRuby2-TUB1 MLC2-mApple* [2μ, *TRP1*, *cyk3*^*TGcΔ*^]), and YEF10371 (*chs1Δ chs3Δ mRuby2-TUB1 MLC2-mApple* [2μ, *TRP1*]). Chitin deposition at PS was visualized by CW (10 μg/mL). n > 80 of division sites were scored. ∗p < 0.01 by Student's t test. See also Figure S4. (F) Effects of deletion of the TGc domain in Cyk3 on septum formation (Chs2-GFP) and AMR constriction (Mlc2-mApple) were determined by imaging of *cyk3*^*TGcΔ*^ strains: YEF9833 (*cyk3*^*TGcΔ*^*CHS2-GFP mRuby2-TUB1 MLC2-mApple*). Kinetics were plotted as bold lines and associated shaded bands that represent mean and SD values, respectively. The reference plots of *CHS2-GFP* in WT and *cyk3*Δ were modified from (C). Montages of division sites were created from selected frames of time-lapse series taken with a 1-min interval of WT (YEF10098) and *cyk3*^*TGcΔ*^ (YEF9833). Scale bars, 1 μm. (G) Effect of deletion of the TGc domain on Cyk3 localization was determined by imaging of WT Cyk3-GFP and *Cyk3*^*TGcΔ*^-GFP strains: YEF9197 and YEF9847 (*cyk3*^*TGcΔ*^*-GFP mRuby2-TUB1 MLC2-mApple*). Kinetics were plotted as bold lines and associated shaded bands that represent mean and SD values, respectively. The reference plots of Myo1-GFP, Chs2-GFP, and GFP-Fks1 in WT were modified from Figures 1E, 2D, and 2F, respectively. Montages of division sites were created from selected frames of time-lapse series taken with a 1-min interval. Scale bars, 1 μm.

Cyk3 is thought to promote PS formation and inhibit precocious SS formation during AMR constriction (Foltman, et al., 2016; Onishi, et al., 2013; Oh, et al., 2012; Meitinger, et al., 2010; Nishihama, et al., 2009; Korinek, et al., 2000). To determine whether and how Cyk3 affects different modules at the system level and to gain additional insights into the mechanism of Cyk3 function, we analyzed the accumulation kinetics of the modular representatives (Myo1-GFP for AMR, Chs2-GFP for PS, and Chs3-GFP and GFP-Fks1 for SS) in *cyk3*Δ cells. We found that deletion of *CYK3* caused various degrees of increased accumulation and delayed removal of all four representatives (Figure 5A), uncovering a role for Cyk3 in AMR constriction and corroborating its roles in PS and SS formation (Foltman, et al., 2016; Meitinger, et al., 2013; Onishi, et al., 2013; Oh, et al., 2012; Meitinger, et al., 2010; Nishihama, et al., 2009; Korinek, et al., 2000). Strikingly, the deletion of *CYK3* impacted only the fast-phase Myo1 constriction, wherein the rate was reduced by ∼40% (Figure 5B). Similar to Myo1-GFP, the accumulation of Chs2-GFP was largely unaffected, but its removal was delayed (Figure 5A). As Cyk3 is a putative activator of Chs2 *in vivo*, and chitin deposition by Chs2 is the major force of ingression (Foltman, et al., 2016; Oh, et al., 2012; Meitinger, et al., 2010; Nishihama, et al., 2009), it is possible that this delay is caused by decreased Chs2 activity in *cyk3*Δ cells. To test this possibility, we examined the impact of several hypomorphic mutations in *CHS2* (I750A, S751A, and N797Q) on its accumulation kinetics. Chs2^I750A^, Chs2^S751A^, and Chs2^N797Q^ reduce the Chs2 activity to ∼75%, ∼45%, and 13.4% of its WT level *in vitro*, respectively (Yabe, et al., 1998). Surprisingly, only Chs2^N797Q^-GFP displayed the slower removal and the failure to trigger the fast-phase AMR constriction (Figures 5C and S4C). Thus, Cyk3 could regulate Chs2 removal and the fast-phase AMR constriction by acting as a major activator of Chs2.

To determine how Cyk3 might activate Chs2, we focused on the transglutaminase core (TGc) domain of Cyk3 (Makarova, et al., 1999). The point mutations at the putative catalytic triad in the TGc domain abolish the ability of Cyk3 to stimulate Chs2 activity (Foltman, et al., 2016). To confirm and extend this observation, we overexpressed either *CYK3* or *cyk3*^*TGc*Δ^, in which the TGc domain (amino acid [aa] 519–580) is deleted, by a multi-copy plasmid in the *chs1*Δ *chs3*Δ strain wherein Chs2 is the sole chitin synthase and the endogenous *CYK3* is present (Oh, et al., 2012). We found that multi-copy *CYK3*, but not *cyk3*^*TGc*Δ^ or the vector control, increased the Chs2-mediated chitin synthesis at the division site (Figure 5E), and also largely rescued the phenotypes of the *chs2*^*N797Q*^*-GFP* cells (Figures 5D and S4D), suggesting that the TGc domain of Cyk3 is critical for its role in Chs2 activation. When the endogenous *CYK3* was replaced with *cyk3*^*TGc*Δ^, defects in Chs2-GFP removal and the fast-phase AMR constriction were observed, although they were milder than those seen in *cyk3*Δ cells (Figure 5F), suggesting that the Cyk3^TGcΔ^ still has some residual activity toward Chs2. Given that a tremendous reduction in Chs2 activity is required to produce any detectable phenotype (Figure S4C), Chs2 activation might be seriously compromised in *cyk3*^*TGc*Δ^ cells. Remarkably, when expressed at the endogenous level, Cyk3^TGcΔ^-GFP was able to localize and constrict during PS formation but disappeared thereafter (Figure 5G). Thus, the TGc domain is essential for Cyk3 localization at the division site only during SS formation. More importantly, this result suggests that the defects in Chs2 activation and AMR constriction in *cyk3*^*TGc*Δ^ cells are caused by a defect in Cyk3 activity, not its localization. Taken together, these data indicate that Cyk3 activates Chs2 via its TGc domain to drive PS formation, which, in turn, drives the switch of the AMR from its slow-phase to fast-phase constriction.

Cyk3 is also thought to prevent precocious SS formation during AMR constriction and PS formation by binding to Rho1 via its TGc domain and inhibiting Rho1 activation (Onishi, et al., 2013). Rho1-GTP directly activates Fks1 (Drgonova, et al., 1996; Qadota, et al., 1996) and also increases the expression of Fks1 and Chs3 via the cell-wall-integrity (i.e., an MAPK) pathway to control the synthesis of β-1,3-glucan and chitin in response to cell wall stress (Jung and Levin, 1999). Consistent with these observations, the accumulation of both GFP-Fks1 and Chs3-GFP was significantly increased in *cyk3*Δ cells (Figure 5A). Strikingly and surprisingly, Cyk3^TGcΔ^-GFP specifically lost its localization during SS formation (Figure 5G), suggesting that the binding of Cyk3 to Rho1 is essential for its localization during SS formation. To evaluate the influence of premature Rho1 activity on other aspects of cytokinesis, we analyzed cells lacking Lrg1, a GAP for Rho1 that specifically acts in the Rho1-Fks1 pathway (Jonasson, et al., 2016). Loss of Lrg1 enhances Rho1 activity and causes premature SS formation (Onishi, et al., 2013; Watanabe, et al., 2001). As expected, the accumulation of both Chs3-GFP and GFP-Fks1 was increased in *lrg1*Δ cells. However, the kinetics of Chs2-GFP and Mlc2-mApple were unaffected (Figures S4A and S4B), suggesting that premature Rho1 activation specifically affects SS formation. Together, these data indicate that Cyk3 inhibits the Fks1 and Chs3 accumulation at the division site not only during PS formation, as demonstrated previously (Onishi, et al., 2013), but also during SS formation presumably by binding to Rho1 via its TGc domain.

In conclusion, the combined kinetic and genetic analyses demonstrate that Cyk3 plays a dual role in cytokinesis by stimulating Chs2-mediated PS formation and inhibiting Fks1- and Chs3-mediated SS formation via its TGc domain. Cyk3-activated PS formation drives the fast-phase AMR constriction, whereas temporally controlled attenuation of the Cyk3-mediated inhibition of Rho1 presumably determines the timing of SS formation.

### Kre6 likely catalyzes β-1,6-glucan synthesis and functions in cell wall maturation between SS formation and cell separation as predicted by its kinetic signature

Proteins functioning in the same module appear to share the same “peak time,” which we designate the “kinetic signature.” For example, proteins involved in PS formation, such as Chs2-GFP and Inn1-GFP, share a kinetic signature (+2 min, Figure 2D), which is different from the one shared by proteins involved in SS formation, such as Chs3-GFP, GFP-Chs4, and GFP-Fks1 (+5 min, Figure 2F). Correlation analysis of the kinetics defined the similarity between modular components (Table S4). For example, Chs2-GFP showed the strongest similarity (1^st^, *r* = 0.95) to Inn1-GFP, whereas Chs3-GFP, GFP-Chs4, and GFP-Fks1 showed weaker similarity (7^th^, 5^th^, and 12^th^; *r* = 0.66, 0.74, and 0.47, respectively). This signature, coupled with genetic analysis, should be able to predict not only the functional time but also the mechanism of an uncharacterized or under-studied protein in cytokinesis.

**Figure 6:**
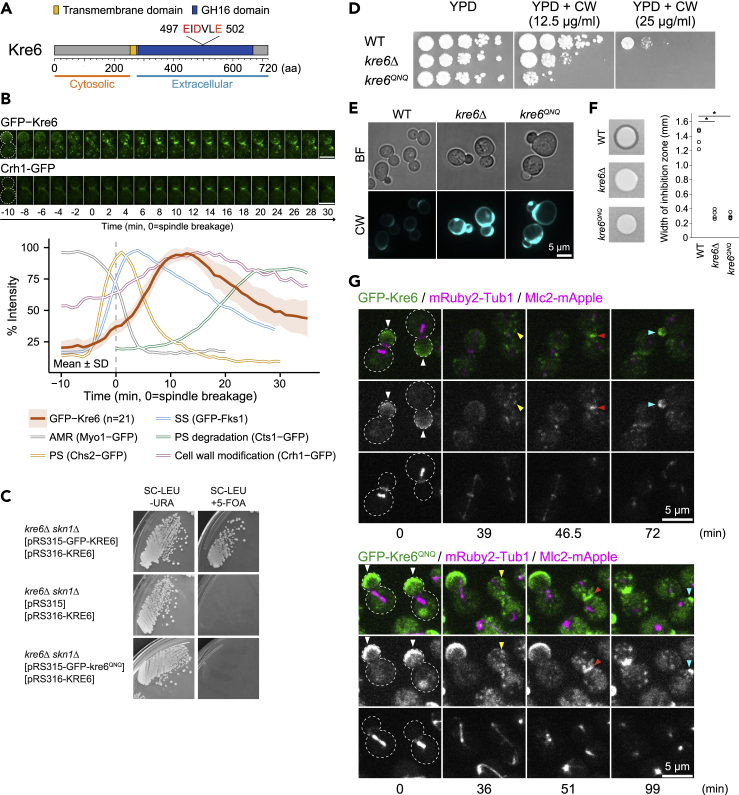
Prediction and validation of Kre6 functioning as a β-1,6-glucan synthase to promote cell wall maturation during cell growth and division (A) Diagram of Kre6. As a type-II membrane protein, Kre6 has an N-terminal cytosolic domain (orange line) and a C-terminal extracellular domain (blue line). The putative catalytic triad of the ExDxxE motif was located in the extracellular GH16 domain (blue box). See also Figure S5. (B) Images of GFP-Kre6 and Crh1-GFP during cytokinesis. Montages of cells were created from selected frames of time-lapse series taken with a 1-min interval. The gray dotted line represents the cell outline. Kinetics were plotted as bold lines and associated shaded bands that represent mean and SD values, respectively. Strains used are as follows: YEF8625 (*GFP-KRE6 mRuby2-TUB1 MLC2-mApple*) and YEF9863 (*CRH1-GFP mRuby2-TUB1 MLC2-mApple*). The reference plots of Myo1-GFP, Chs2-GFP, and GFP-Fks1 in WT were modified from Figures 1E, 2D, and 2F, respectively. All strains were cultured in SC medium except for YEF9863, which was cultured in neutralized SC medium. Scale bars, 5 μm. See also Table S5. (C) Mutations in the ExDxxE motif (QNQ-mutation) disrupt the functionality of Kre6. Growth of cells carrying *LEU2*-plasmid of WT *KRE6* (pRS315-GFP-KRE6), empty vector (pRS315), and *kre6*^*QNQ*^ (pRS315-GFP-kre6^QNQ^) are tested on plates containing the medium SC-LEU-URA or SC-LEU+5-FOA to selecting for or against the cover plasmid (pRS316-KRE6), respectively. Strains used are as follows: YEF10141 (*kre6Δ skn1Δ* [CEN, *URA3*, *KRE6*] [CEN, *LEU2*, *GFP*-*KRE6*]), YEF10143 (*kre6Δ skn1Δ* [CEN, *URA3*, *KRE6*] [CEN, *LEU2*]), and YEF10142 (*kre6Δ skn1Δ* [CEN, *URA3*, *KRE6*] [CEN, *LEU2*, *GFP*-*kre6*^*QNQ*^]). Cells were incubated at 25°C for 4 days. See also Figure S5. (D–F) The *kre6*^*QNQ*^ allele causes cell wall defects. (D) The sensitivity of WT, *kre6*Δ, and *kre6*^*QNQ*^ cells to CW was tested on YPD plates containing CW. Cells were incubated at 25°C for 6 days. (E) The chitin deposition in the cell wall of WT, *kre6*Δ, and *kre6*^*QNQ*^ cells. Cells cultured to exponentially phase in SC medium at 25°C were resuspended in SC medium containing CW (10 μg/mL) to stain the cell wall chitin. (F) The K1 killer toxin sensitivity of WT, *kre6*Δ, and *kre6*^*QNQ*^ was tested by measuring the growth inhibition to the toxin-producing strain. Growth of testing yeast lawn was recorded after 3 days of incubation at 25°C. Strains used are as follows: YEF10248 (WT *KRE6*), YEF10249 (*kre6*Δ), YEF10250 (*kre6*^*QNQ*^), and NCYC232 (toxin-producing strain). See also Figure S5. (G) Images of GFP-tagged Kre6 and Kre6^QNQ^ during the cell cycle. Both GFP-Kre6 and GFP-Kre6^QNQ^ were accumulated at the bud cortex in G2/M phase (white arrowheads). They were internalized as puncta (yellow arrowheads) at the onset of cytokinesis and then localized to the bud neck (red arrowheads). In the new cell cycle, Kre6 was re-localized to the bud cortex (cyan arrowheads). Strains used are as follows: YEF8625 (*GFP-KRE6 mRuby2-TUB1 MLC2-mApple*) and YEF10337 (*GFP-KRE6*^*QNQ*^*mRuby2-TUB1 MLC2-mApple*). Montages of cells were created from selected frames of time-lapse series taken with a 1.5-min interval. The gray dotted line represents the cell outline.

Genetic evidence indicates that Kre6, a type-II membrane protein (Figure 6A), is involved in the synthesis of β-1,6-glucan (Roemer, et al., 1993; Roemer and Bussey, 1991), an essential component of the fungal cell walls, but when, where, and how it functions remains poorly understood. We hypothesize that Kre6 is involved in cytokinesis (separation of cytoplasm between mother and daughter cells) or cell separation (detachment between mother and daughter cells) for the following reasons: (1) *kre6*Δ cells show a multiple bud phenotype, indicative of a defect in cytokinesis or cell separation (Dijkgraaf, et al., 2002), and (2) Sbg1, the Kre6 ortholog in fission yeast, is involved in cytokinesis by coupling AMR constriction to PS formation (Davidson, et al., 2016; Sethi, et al., 2016). *KRE6* has a paralog, *SKN1*, in budding yeast, but we focused our analysis on *KRE6* due to its clear involvement in β-1,6-glucan synthesis (Roemer, et al., 1993). We constructed GFP-Kre6 and confirmed that this allele was functional, as it suppressed the lethality of *kre6*Δ *skn1*Δ (Figures S5A and S5B). Time-lapse analysis indicated that GFP-Kre6 localized to the bud cortex, which is consistent with the previous immunostaining result (Kurita, et al., 2012). GFP-Kre6 also showed bud neck localization, which was not reported previously (Figure 6B). Strikingly, GFP-Kre6 peaked between SS formation and PS degradation, which is unique among all the proteins examined thus far (Figure 6B). Because Kre6 is involved in β-1,6-glucan synthesis (Roemer, et al., 1993) and the transglycosylase Crh1 attaches chitin to both β-1,6-glucan and β-1,3-glucan (Cabib, 2009), we also examined the kinetics of Crh1-GFP (GFP inserted after residue 56 in Crh1, Rodriguez-Pena, et al., 2000). Remarkably, Crh1-GFP peaked significantly after Chs3-GFP and GFP-Fks1 and slightly after GFP-Kre6, which is perfectly consistent with its role in cross-linking the products of these synthetic enzymes (Figure 6B). Correlation analysis of the kinetics between GFP-Kre6 and other proteins revealed that Kre6 and Crh1 showed the strongest similarity among proteins analyzed (1^st^, *r* = 0.96, Table S5). Together, these data suggest that Kre6 functions at the division site between SS formation and cell separation and is unlikely to be involved in the coupling of the AMR to PS formation as suggested for its counterpart in fission yeast (Davidson, et al., 2016; Sethi, et al., 2016).

To test whether Kre6 is involved in cytokinesis, we screened for temperature-sensitive mutations in *KRE6* by random mutagenesis in a *skn1*Δ background and identified two alleles (*kre6*^*N461S*^ *skn1*Δ and *kre6*^*E582G*^ *skn1*Δ). Both mutants showed temperature sensitivity for growth at 37°C on plate or in liquid medium (Figures S5C and S5D). Both mutants displayed some cell clumps or clusters at the non-permissive temperature, indicative of a defect in cytokinesis and/or cell separation. However, upon mild sonication (to reduce clumps for imaging), neither mutant showed a multiple bud phenotype, suggesting no apparent defect in cytokinesis (Figures S5E and S5F), although it remains possible that a weak defect in cell separation in the mutant cells was abolished by sonication. Both mutants exhibited the stereotypical phenotypes for cells with defective cell walls, including broad-neck morphology, sensitivity to CW, enhanced chitin deposition into the cell wall, and lethality suppression by the osmotic support of 1 M sorbitol (Figures S5E and S5G–S5I) (Kubo, et al., 2019; Levin, 2011; Ram and Klis, 2006). These data demonstrate that, in contrast to its fission yeast ortholog Sbg1, Kre6 is not involved in the AMR and PS-mediated membrane closure, and that its primary function is likely to be in the cell wall biogenesis or maturation. This difference in function might reflect the fact that Sbg1 is shorter than Kre6, containing only the N-terminal transmembrane domain of Kre6, but lacking the C-terminal glycosyl hydrolase family 16 (GH16) domain (Davidson, et al., 2016; Sethi, et al., 2016), which is likely involved in β-1,6-glucan synthesis (see below).

Kre6 has the hallmarks of glycoside hydrolases or transglycosidases (Montijn, et al., 1999) and is required for β-1,6-glucan synthesis (Roemer, et al., 1993), but whether it functions as the catalytic enzyme remains unclear. To address this question, we analyzed its domain function. Kre6 belongs to the GH16 family, which includes Crh1 that catalyzes chitin-glucan cross-linking and hence is considered a cell wall maturation enzyme (Cabib, 2009). GH16 proteins have a consensus motif (ExDxE or ExDxxE) for their catalytic activity (Blanco, et al., 2015). We found a similar motif in Kre6 (ExDxxE at aa 497-502). To examine its function, we mutated three residues to QxNxxQ (hereafter called *kre6*^*QNQ*^), and the *kre6*^*QNQ*^ allele failed to suppress the lethality of *kre6*Δ *skn1*Δ (Figure 6C), suggesting that these residues are essential for its function. The *kre6*^*QNQ*^ strain exhibited defective cell wall phenotypes, such as increased sensitivity to CW and increased chitin deposition in the cell wall (Figures 6D and 6E). Because the deletion of *KRE6* leads to a reduction in the amount of β-1,6-glucan in the cell wall (Roemer, et al., 1993), we assessed β-1,6-glucan content by testing for sensitivity to the K1 killer toxin, as the toxin requires β-1,6-glucan for its incorporation and subsequent formation of lethal pores on the membrane (Roemer, et al., 1993). As expected, the *kre6*^*QNQ*^ and *kre6*Δ strains were resistant to K1 killer toxin (Figure 6F), suggesting that the β-1,6-glucan content was reduced. This result demonstrates that the ExDxxE motif in Kre6 is essential for its function, likely by catalyzing β-1,6-glucan synthesis.

Kre6 is thought to participate in β-1,6-glucan synthesis at the Golgi (Shahinian and Bussey, 2000) or at the PM (Montijn, et al., 1999). To gain additional insight into this question, we performed time-lapse analysis on Kre6 localization during the cell cycle. We found that Kre6 accumulated at the bud cortex (white arrowheads), was internalized into endosome-like puncta (yellow arrowheads) precisely at the time of spindle breakage, and was then re-localized to the bud neck (red arrowheads) (Figure 6G). After cytokinesis and cell separation, Kre6 was re-localized to the new bud cortex (cyan arrowheads) (Figure 6G). These data suggest that Kre6 is more likely to function at the PM, providing an additional support to one of the previous studies (Montijn, et al., 1999). We also found that GFP-Kre6^QNQ^ exhibited a similar pattern of localization to the WT protein (Figure 6G), suggesting that the catalytic residues are dispensable for proper localization but essential for β-1,6-glucan synthesis at the PM. Of note, the intensity of GFP-Kre6^QNQ^ was higher than that of GFP-Kre6, which might be caused by increased expression of *KRE6* as the result of a cell wall stress response and/or prolonged binding between an inactive enzyme and its substrate at its functional location.

In conclusion, our kinetic analysis makes and then validates the prediction that Kre6 functions temporally between SS formation and cell separation at the division site and is not involved in AMR-PS coupling as its counterpart does in fission yeast. Furthermore, our genetic analysis on the putative catalytic motif strongly suggests that Kre6 is the enzyme responsible for β-1,6-glucan synthesis at the sites of polarized cell growth to drive cell wall biogenesis and maturation.

## Discussion

**Figure 7:**
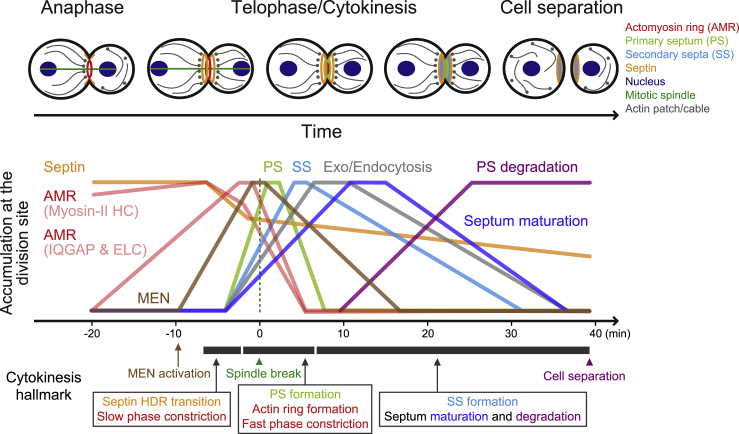
The kinetic landscape of cytokinesis As the cell cycle progresses from the onset of mitotic exit to cell separation, distinct functional modules accumulate at the division site in a strict temporal order to play their specific roles in cytokinesis. The kinetics were modified from Figures 1, 2, 3, and 6. See text for details.

Cytokinesis is a complex process involving the interplay of many conserved proteins, but the temporal and kinetic relationships between these proteins at the division site have not been comprehensively analyzed under the same experimental setting using the same cell cycle clock, with the exception of a temporal analysis of cytokinetic proteins in fission yeast (Wu, et al., 2003). In this study, we performed quantitative live-cell imaging to analyze the accumulation kinetics of more than 20 core cytokinetic proteins at the division site from five distinct modules to depict the kinetic landscape of cytokinesis at the system level (Figure 7). This imaging strategy, coupled with genetic or chemical perturbations, has revealed connections between different modules and provided mechanistic insights into the biphasic constriction of the AMR and the roles of F-actin and the transglutaminase-like protein Cyk3 in cytokinesis. Importantly, we found that the kinetic signature defined in this study can be used as a predictive parameter, coupled with functional analysis, to define the role of an uncharacterized protein, such as the type-II membrane protein Kre6, in cytokinesis.

### Kinetic analysis reveals the temporal order of cytokinetic modules and identifies the likely drivers for the switch from the slow- to fast-phase constriction

Kinetic analysis of all the representatives from different modules indicates that these modules function at the division site in a strict order (Figure 7). During anaphase, a component of the MEN, the temporal cue for cytokinesis, arrives at the bud neck (-10 min). A few minutes later, septins undergo the HDR transition that coincides with the onset of AMR constriction (-6 min). The constriction is initially slow, and then ramped up (from ∼-2 min) to complete cytokinesis. The switch from the slow- to fast-phase constriction coincides with the completion of the septin HDR, the initial recruitment of exo-endocytic components, and the peaking of the AMR (IQGAP and ELC), PS formation (Chs2), and MEN (Cdc14) components. These observations suggest that the completion of the HDR transition clears the septin filaments between the AMR and the PM, which enables the anchoring of the AMR to the PM and permits its efficient constriction (Chen, et al., 2020; Tamborrini, et al., 2018). These observations also suggest that AMR maturation and ring-guided PS formation likely drive the fast-phase constriction. AMR constriction is followed sequentially by PS formation (Chs2), SS formation (Chs3 and Fks1), septum maturation (Crh1 and Kre6), and eventually cell separation (Cts1). Thus, this comprehensive analysis has not only revealed the temporal order of cytokinetic modules but also identified the likely mechanisms underlying the switch from the slow- to fast-phase constriction.

### Cyk3 plays distinct roles in PS and SS formation and controls the fast-phase AMR constriction

Kinetic and genetic analyses have uncovered the distinct roles of the TGc domain of Cyk3 in PS and SS formation as well as the role of Cyk3 in controlling the fast-phase ring constriction. Chs2 plays a pivotal role in cytokinesis by driving PS formation. Chs2 appears to be inactive at the delivery and then activated on-site by an unknown mechanism. Inn1 and Cyk3 have been implicated in Chs2 activation, as Inn1 is essential and Cyk3 is partially required for PS formation (Wang, et al., 2018; Foltman, et al., 2016; Devrekanli, et al., 2012; Meitinger, et al., 2010; Nishihama, et al., 2009). How Cyk3 activates Chs2 to promote PS formation remains unclear. It was proposed that Inn1 interacts with Chs2 via its C-terminus at the division site to prevent the precocious activation of Chs2 (Foltman, et al., 2016). Upon the arrival of Cyk3 at the division site, Inn1 interacts with the N-terminal SH3 domain of Cyk3 via a PXXP motif in its C terminus (Nishihama, et al., 2009), thus relieving its C-terminus-mediated inhibition of Chs2 (Foltman, et al., 2016). Cyk3 may also bind directly to Chs2 via multiple domains, including its central TGc domain (Foltman, et al., 2016). The TGc domain in Cyk3 is unconventional in that it lacks a conserved cysteine in the putative catalytic triad (Makarova, et al., 1999), whereas this TGc domain and other conserved residues within it are essential for the Cyk3 dosage-dependent activation of Chs2 *in vivo* (Foltman, et al., 2016) (this study). Strikingly, our kinetic analysis indicates that Cyk3 lacking the TGc domain localizes to the division site normally during PS formation but disappears from the division site specifically during SS formation. In addition, this mutant allele of *CYK3* as well as the hypomorphic allele of *CHS2* compromise PS formation and the switch of AMR constriction from its slow phase to its fast phase. Together, these observations strongly suggest that the TGc domain of Cyk3 affects the activation of Chs2 at the division site, and that PS formation contributes to the switch from the slow- to fast-phase constriction. Chs2 requires proteolysis for its activation *in vitro* (Martinez-Rucobo, et al., 2009; Uchida, et al., 1996; Sburlati and Cabib, 1986). Interestingly, the TGc domains of a bacteriophage protein and a mouse muscular dystrophy protein possess protease activity (Beatham, et al., 2004; Blanco, et al., 2001; Pfister, et al., 1998). Thus, it is possible that Cyk3 activates Chs2 *in vivo* by acting as a protease via its TGc domain. Taken together, two distinct domains of Cyk3 (SH3 and TGc) appear to be involved in Chs2 activation *in vivo*, but their relative contributions and detailed mechanisms of action require further investigation.

Our kinetic analysis has also revealed that the TGc domain is essential for Cyk3 localization at the division site, specifically during SS formation. This localization is likely controlled by the interaction between Rho1 and the TGc domain of Cyk3 (Onishi, et al., 2013), which presumably ensures the appropriate timing and rate for Rho1 activation and its role in promoting SS formation via Fks1 and Chs3. Importantly, the role of Cyk3 in Rho1-mediated SS formation is likely independent of its role in PS formation, as deletion of Lrg1, a GAP for Rho1 that functions during SS formation (Jonasson, et al., 2016; Onishi, et al., 2013; Watanabe, et al., 2001), does not affect the kinetics of Chs2 accumulation at the division site or the rate of AMR constriction. Collectively, these observations suggest that Cyk3 controls the switch of AMR constriction from its slow phase to its fast phase via its role in PS formation.

### Kre6 likely catalyzes β-1,6-glucan synthesis at the cell surface to promote cell wall maturation during cell growth and division

Our prediction based on kinetic signature, followed by validation experiments, has led to the discovery of a critical role of Kre6 in cell wall maturation between SS formation and cell separation. This role likely involves the synthesis of β-1,6-glucan by Kre6 at the cell surface that is branched off the Fks1-synthesized β-1,3-glucan. β-1,6-glucan is an essential component of the cell wall, interconnecting other wall components, including cell wall proteins, β-1,3-glucan, and chitin (Shahinian and Bussey, 2000; Montijn, et al., 1999). However, three fundamental questions regarding β-1,6-glucan synthesis remained elusive: the identity and functional location of its synthase and the coordination of β-1,6- and β-1,3-glucan synthesis during cell wall assembly. Deletion of *KRE6* is known to cause a reduction in β-1,6-glucan content, which leads to resistance to K1 killer toxin (Roemer, et al., 1994; Roemer, et al., 1993; Roemer and Bussey, 1991). Given that Kre6 contains a GH16 domain, the hallmark of glycoside hydrolases or transglycosidases (Montijn, et al., 1999), we mutagenized its putative catalytic motif (ExDxxE) and found that the mutant allele did not affect Kre6 localization, but completely failed to rescue the killer toxin resistance. Thus, Kre6 is highly likely to possess the enzymatic activity required for β-1,6-glucan synthesis. Our time-lapse indicates that Kre6 first localizes to the bud membrane without any accumulation in an intracellular organelle and then relocalizes, through endocytosis, to the bud neck during cytokinesis. This observation suggests that Kre6 likely catalyzes the synthesis of β-1,6-glucan at the PM, which corroborates a previous conclusion (Montijn, et al., 1999).

Strikingly, Kre6 peaks after Fks1 at the division site, suggesting that β-1,6-glucan synthesis may occur after β-1,3-glucan. Fks1 appears to only synthesize linear β-1,3-glucan (Chhetri, et al., 2020). Interestingly, mutations in *FKS1* affect both types of glucan synthesis, suggesting that the linear β-1,3-glucan polymer might serve as the acceptor for the β-1,6-glucan branching (Dijkgraaf, et al., 2002). Both Kre6 and Fks1 display a similar localization pattern during the cell cycle (Kurita, et al., 2012; Utsugi, et al., 2002), and deletion of *KRE6* causes delocalization of Fks1 from the PM (Dijkgraaf, et al., 2002). Collectively, these observations suggest that Kre6 might coordinate both types of glucan synthesis at the cell surface by catalyzing the formation of β-1,6-glucan that branches off the linear β-1,3-glucan polymer to promote cell wall biogenesis or maturation during cell growth and division.

In fission yeast, Sbg1, the ortholog of the budding yeast Kre6, is required for cytokinesis by regulating the localization of Bgs1, a β-1,3-glucan synthase that is essential for PS formation (Davidson, et al., 2016; Sethi, et al., 2016). Because of the fundamental difference in PS formation between the budding yeast (Chs2-controlled chitin synthesis) and the fission yeast (Bgs1-controlled β-1,3-glucan synthesis), the functions of Kre6 in budding yeast and its counterpart Sbg1 in fission yeast during cytokinesis are superficially quite different. However, at the mechanistic level, the difference is much smaller than it appears. Sbg1 contains only the N-terminal counterpart of Kre6, including the transmembrane domain, but lacks the GH16 domain (Sethi, et al., 2016). Not surprisingly, the β-1,6-glucan content is not affected in the *sbg1* mutant (Sethi, et al., 2016). Thus, Sbg1 can only carry out one of two functions for Kre6, controlling the localization of a β-1,3-glucan synthase at the PM but not catalyzing the synthesis of β-1,6-glucan. We searched the genome database of *S. pombe* and found two other uncharacterized genes (*SPAC23H3.11c* and *SPAC17G6.11c*) that would encode proteins with similar sequence and domain organization, including the GH16 domain, as in Kre6. It would be very interesting to determine whether these two proteins promote cell wall maturation during cell growth and division by controlling β-1,6-glucan synthesis off the linear β-1,3-glucan polymer as suggested for Kre6 in budding yeast.

### Limitations of the study

Our study has established a kinetic landscape of protein networks involved in cytokinesis, revealed the order and interplay between different functional modules, and provided further mechanistic insights into the functions of specific proteins. Despite this significant progress, biochemical study is required to further solidify some of the conclusions that are drawn mainly based on genetic and cell biological arguments as well as on existing data. For example, we show here that the TGc domain of Cyk3 is essential for its localization at the division site during SS formation, but we do not know whether the interaction of the TGc with Rho1 or other cytokinetic protein(s) is responsible for this localization. Our analysis and existing data also suggest that Kre6 and Fks1 might coordinate the synthesis of β-1,6-glucan and β-1,3-glucan at the sites of polarized cell growth, but the coordination mechanism at the biochemical level remains unknown.

### Resource availability

#### Lead contact

Further information and requests for resources and reagents should be directed to and will be fulfilled by the Lead Contact, Erfei Bi (ebi@pennmedicine.upenn.edu).

#### Materials availability

Reagents generated in this study will be made available upon request.

#### Data and code availability

Data supporting the findings of this study are available within the paper and its Supplemental information, and also from the authors upon request.

## Methods

All methods can be found in the accompanying Transparent methods supplemental file.

## Supplemental information

Document S1. Transparent methods, Figures S1–S5, and Tables S1–S5

## Footnotes

Supplemental Information can be found online at https://doi.org/10.1016/j.isci.2020.101917.
