# Supplementary material for: The kinetic landscape and interplay of protein networks in cytokinesis
Source: iScience. 2020 Dec 11;24(1):101917. doi: 10.1016/j.isci.2020.101917 (PMC7773586; doi:10.1016/j.isci.2020.101917)
Supplement: Document S1. Transparent methods, Figures S1–S5, and Tables S1–S5 [file mmc1.pdf]

iScience, Volume *24*

Supplemental Information

The kinetic landscape and interplay of protein networks in cytokinesis

Hiroki Okada, Brittany MacTaggart, Yoshikazu Ohya, and Erfei Bi

SEPPLEMENTAL FIGURES

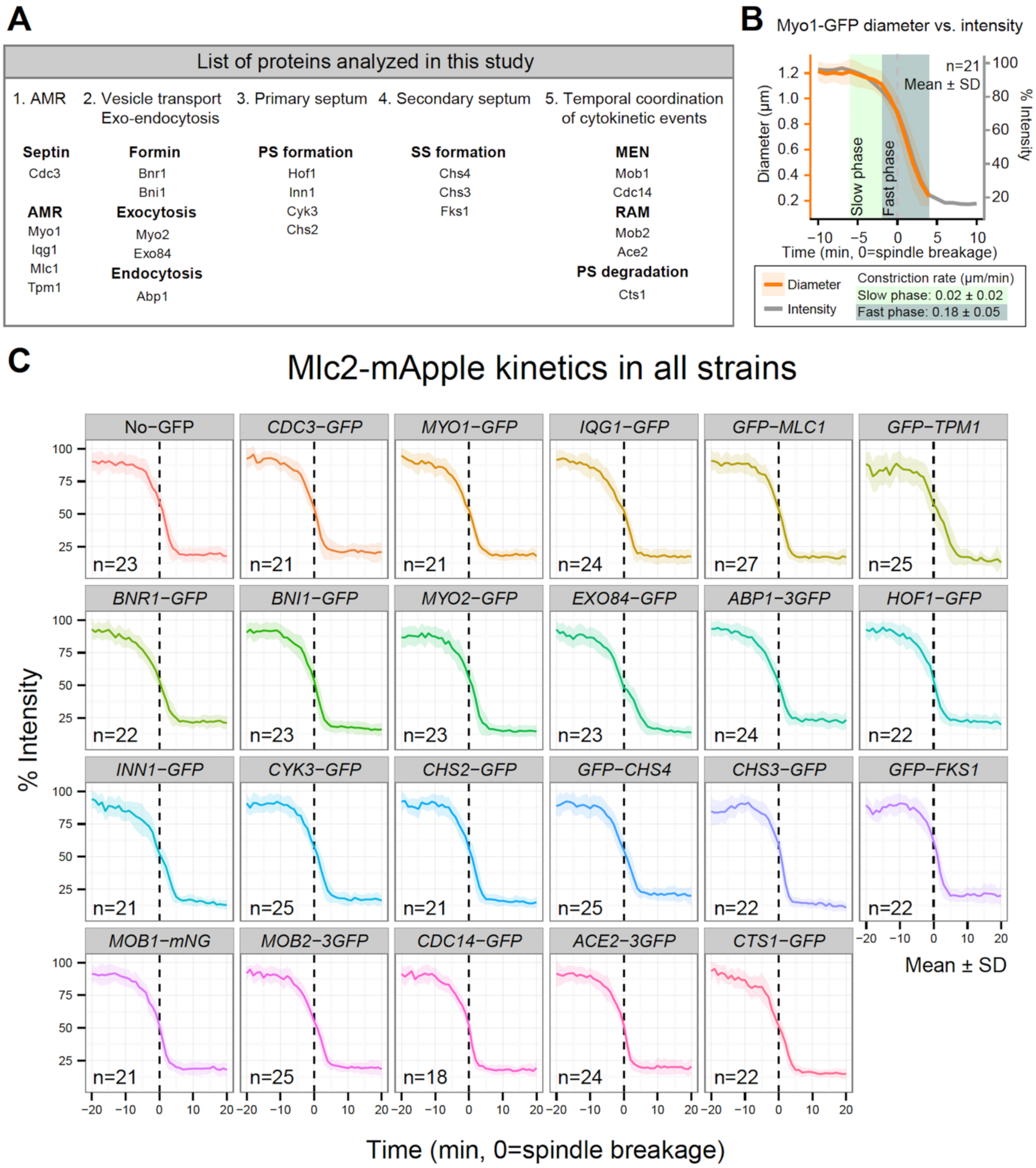

Figure S1. The core cytokinetic proteins analyzed in this study. Related to Figure 1.

(A) List of proteins analyzed in this study. Twenty-two proteins were selected from all aspects of cytokinesis. See text for details.

(B) Diameter and intensity of Myo1-GFP during constriction. Plots were created from the data in **Figures 1B and 1D**.

(C) The individual plots of the kinetics of Mlc2-mApple from all strains. See also **Figure 1C**. Bold lines and associated shaded bands represent mean and SD values, respectively.

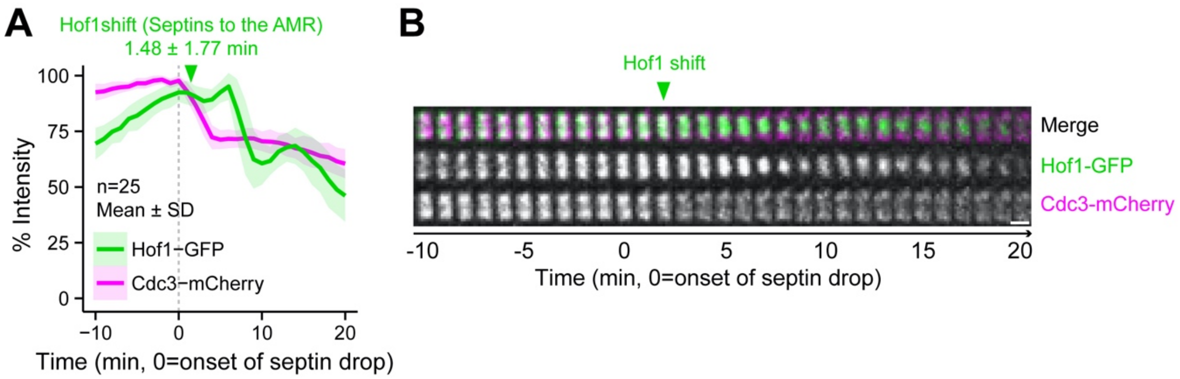

Figure S2. The kinetics and localization change of Hof1 during the septin HDR transition. Related to Figure 2.

(A) Kinetics of Hof1-GFP and Cdc3-mCherry. The strain used is YEF8631 (WT *HOF1-GFP CDC3-mCherry*). Bold lines and associated shaded bands represent mean and SD values, respectively.

(B) Montages of the division site were created from selected frames of time-lapse series taken with a 1-min interval presented in (A). The shift of Hof1-GFP from the mother side of the septin hourglass to the middle of the division site occurred at or immediately after the onset of septin HDR. Scale bar, 1 μm.

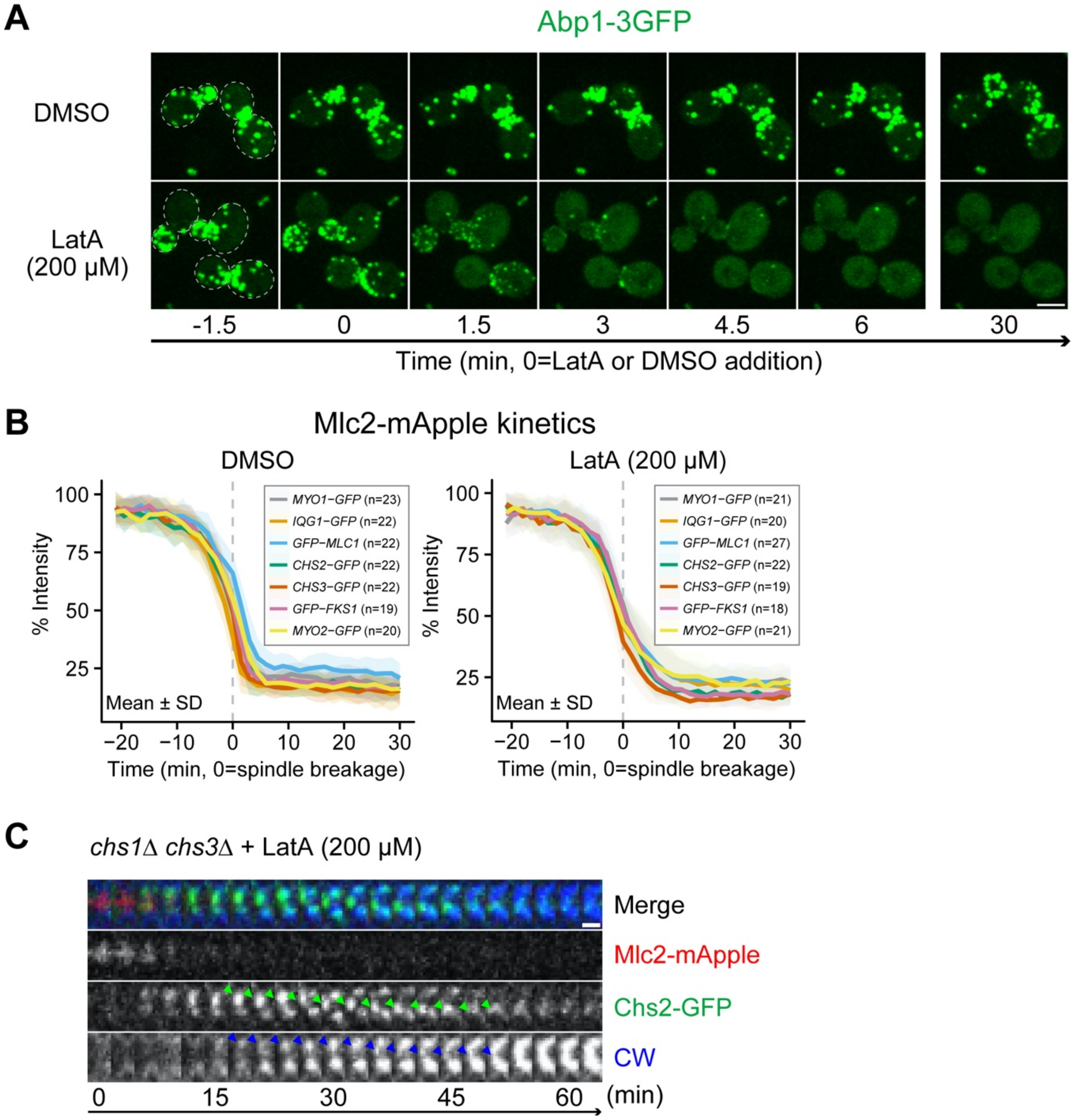
A
Abp1-3GFP
DMSO
LatA
(200 μM)
-1.5
0
1.5
3
4.5
6
30
Time (min, 0=LatA or DMSO addition)
B
Mlc2-mApple kinetics
DMSO
LatA (200 μM)
% Intensity
MYO1−GFP (n=23)
IQG1−GFP (n=22)
GFP−MLC1 (n=22)
CHS2−GFP (n=22)
CHS3−GFP (n=22)
GFP−FKS1 (n=19)
MYO2−GFP (n=20)
MYO1−GFP (n=21)
IQG1−GFP (n=20)
GFP−MLC1 (n=27)
CHS2−GFP (n=22)
CHS3−GFP (n=19)
GFP−FKS1 (n=18)
MYO2−GFP (n=21)
Mean ± SD
Time (min, 0=spindle breakage)
C
chs1Δ chs3Δ + LatA (200 μM)
Merge
Mlc2-mApple
Chs2-GFP
CW
0
15
30
45
60
(min)

Figure S3. Effect of latrunculin A on actin patches, AMR, and PS formation. Related to Figure 4.

(A) The disruption of F-actin by LatA (200 µM) was determined by imaging a mixed culture of the strain YEF9198 (*ABP1-3GFP*) and another strain expressing a GFP-tagged cytokinetic protein [e.g., YEF9609 (*MYO1-GFP*)]. Disappearance of Abp1-3GFP patches was observed within ~5 min after LatA addition. Scale bar, 3 µm.

(B) Kinetics of Mlc2-mApple from all strains in the presence of DMSO or LatA. Data were acquired from the time-lapse imaging presented in **Figure 4**. Bold lines and associated shaded bands represent mean and SD values, respectively.

(C) Montages of the division site were created from selected frames of time-lapse series taken with a 1.5-min interval. The strain used is YEF10368 (*chs1*Δ *chs3*Δ *CHS2-GFP-TRP1 mRuby2-TUB1 MLC2-mApple*). Cells cultured to exponentially phase in SC medium at 25°C were resuspended in SC medium containing LatA (200 µM) and CW (10 µg/ml) to visualize the impact of F-actin disruption on PS formation. Scale bar, 1 µm.

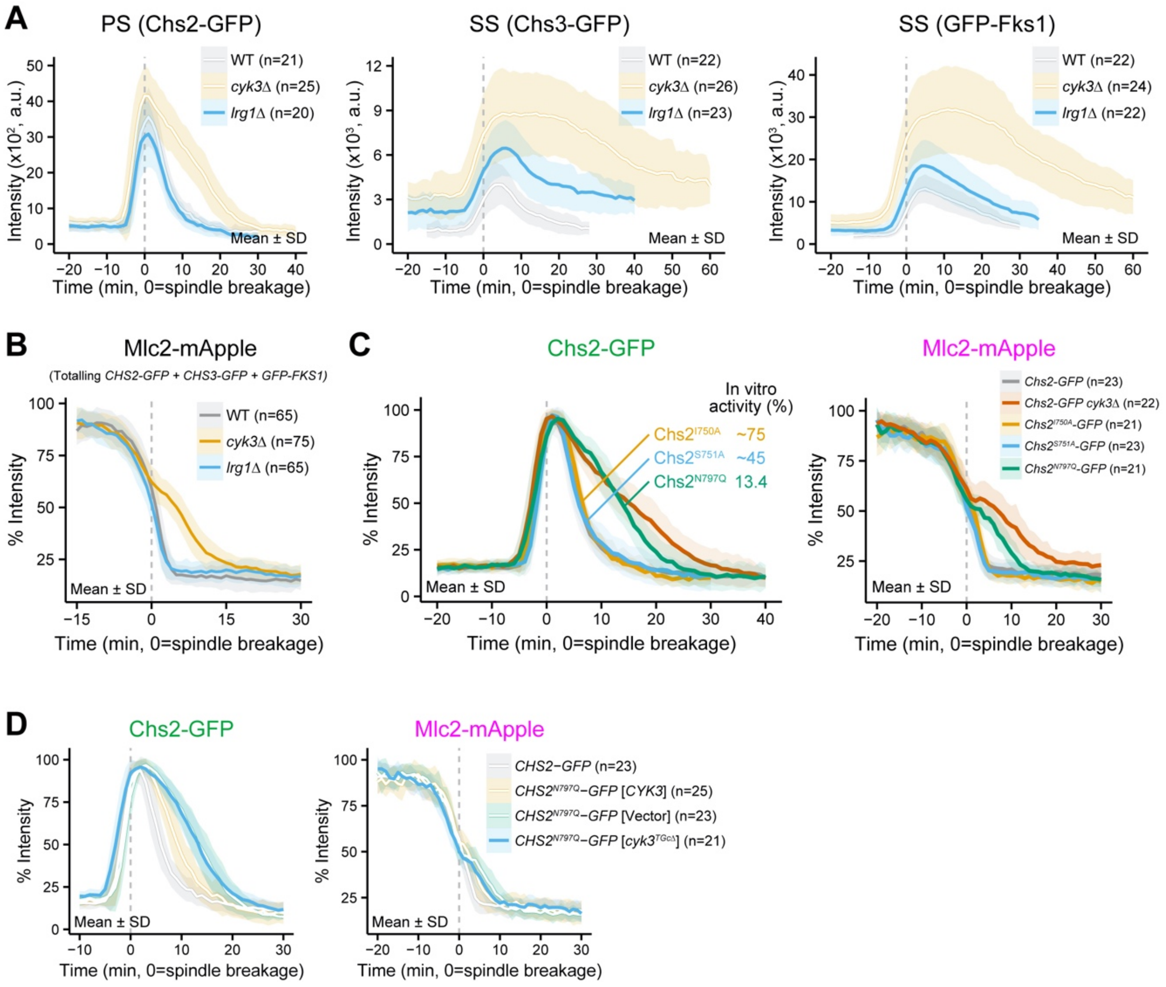

A
PS (Chs2-GFP)
WT (n=21)
cyk3Δ (n=25)
lrg1Δ (n=20)
Intensity (x10², a.u.)
Mean ± SD
Time (min, 0=spindle breakage)
SS (Chs3-GFP)
WT (n=22)
cyk3Δ (n=26)
lrg1Δ (n=23)
Intensity (x10³, a.u.)
Mean ± SD
Time (min, 0=spindle breakage)
SS (GFP-Fks1)
WT (n=22)
cyk3Δ (n=24)
lrg1Δ (n=22)
Intensity (x10³, a.u.)
Mean ± SD
Time (min, 0=spindle breakage)
B
Mlc2-mApple
(Totalling CHS2-GFP + CHS3-GFP + GFP-FKS1)
WT (n=65)
cyk3Δ (n=75)
lrg1Δ (n=65)
% Intensity
Mean ± SD
Time (min, 0=spindle breakage)
C
Chs2-GFP
In vitro activity (%)
Chs2I750A ~75
Chs2S751A ~45
Chs2N797Q 13.4
% Intensity
Mean ± SD
Time (min, 0=spindle breakage)
Mlc2-mApple
Chs2-GFP (n=23)
Chs2-GFP cyk3Δ (n=22)
Chs2I750A-GFP (n=21)
Chs2S751A-GFP (n=23)
Chs2N797Q-GFP (n=21)
% Intensity
Mean ± SD
Time (min, 0=spindle breakage)
D
Chs2-GFP
% Intensity
Mean ± SD
Time (min, 0=spindle breakage)
Mlc2-mApple
CHS2−GFP (n=23)
CHS2N797Q−GFP [CYK3] (n=25)
CHS2N797Q−GFP [Vector] (n=23)
CHS2N797Q−GFP [cyk3TGcΔ] (n=21)
% Intensity
Mean ± SD
Time (min, 0=spindle breakage)

Figure S4. Effect of *LRG1* deletion, *chs2* hypomorphic mutations, and overexpression of *CYK3*$^{TGc\Delta}$ on PS formation and AMR. Related to Figure 5.

(A) Effects of precocious Rho1 activation on PS and SS formation were determined by imaging of *lrg1*Δ strains: YEF10291 (*lrg1*Δ *CHS2-GFP mRuby2-TUB1 MLC2-mApple*), YEF10292 (*lrg1*Δ *CHS3-GFP mRuby2-TUB1 MLC2-mApple*), and YEF10290 (*lrg1*Δ *GFP-FKS1 mRuby2-TUB1 MLC2-mApple*). Kinetics were plotted as bold lines and associated shaded bands that represent mean and SD values, respectively. The reference plots of Chs2-GFP, Chs3-GFP, and GFP-Fks1 in WT and *cyk3*Δ were modified from **Figure 5A**.

(B) Effect of precocious Rho1 activation on AMR (Mlc2-mApple). Data were acquired from the time-lapse imaging presented in (A) and **Figure 4A**.

(C) Effects of hypomorphic mutations of *CHS2* in comparison to *cyk3*Δ on PS formation were determined by imaging of strains: YEF10098 (*CHS2-GFP mRuby2-TUB1 MLC2-mApple*), YEF10153 (*cyk3*Δ *CHS2-GFP mRuby2-TUB1 MLC2-mApple*), YEF10443 (*chs2*^{I750A}*-GFP mRuby2-TUB1 MLC2-mApple*), YEF10100 (*chs2*^{S751A}*-GFP mRuby2-TUB1 MLC2-mApple*), and YEF10101 (*chs2*^{N797Q}*-GFP mRuby2-TUB1 MLC2-mApple*). Kinetics were plotted as bold lines and associated shaded bands that represent mean and SD values, respectively. Mutations of I750A, S751A, and N797Q reduce the Chs2 activity to ~75, ~45, and 13.4% of its wild-type level in vitro (Yabe, et al., 1998). See also **Figure 5C**.

(D) Effects of overexpression of *CYK3*$^{TGc\Delta}$ on septum formation (Chs2-GFP) and AMR constriction (Mlc2-mApple) in *chs2*^{N797Q} cells were determined by imaging the cells carrying *CYK3*$^{TGc\Delta}$ high-copy plasmid: YEF10378 (*chs2*^{N797Q}*-GFP mRuby2-TUB1 MLC2-mApple* [2μ, *TRP1*, *CYK3*$^{TGc\Delta}$]). The reference plots of *CHS2-GFP* and *chs2*^{N797Q}*-GFP* with overexpression of *CYK3* (*chs2*^{N797Q}*-GFP* [*CYK3*]) and empty vector (*chs2*^{N797Q}*-GFP* [Vector]) were modified from **Figure 5D**. Kinetics were plotted as bold lines and associated shaded bands that represent mean and SD values, respectively.

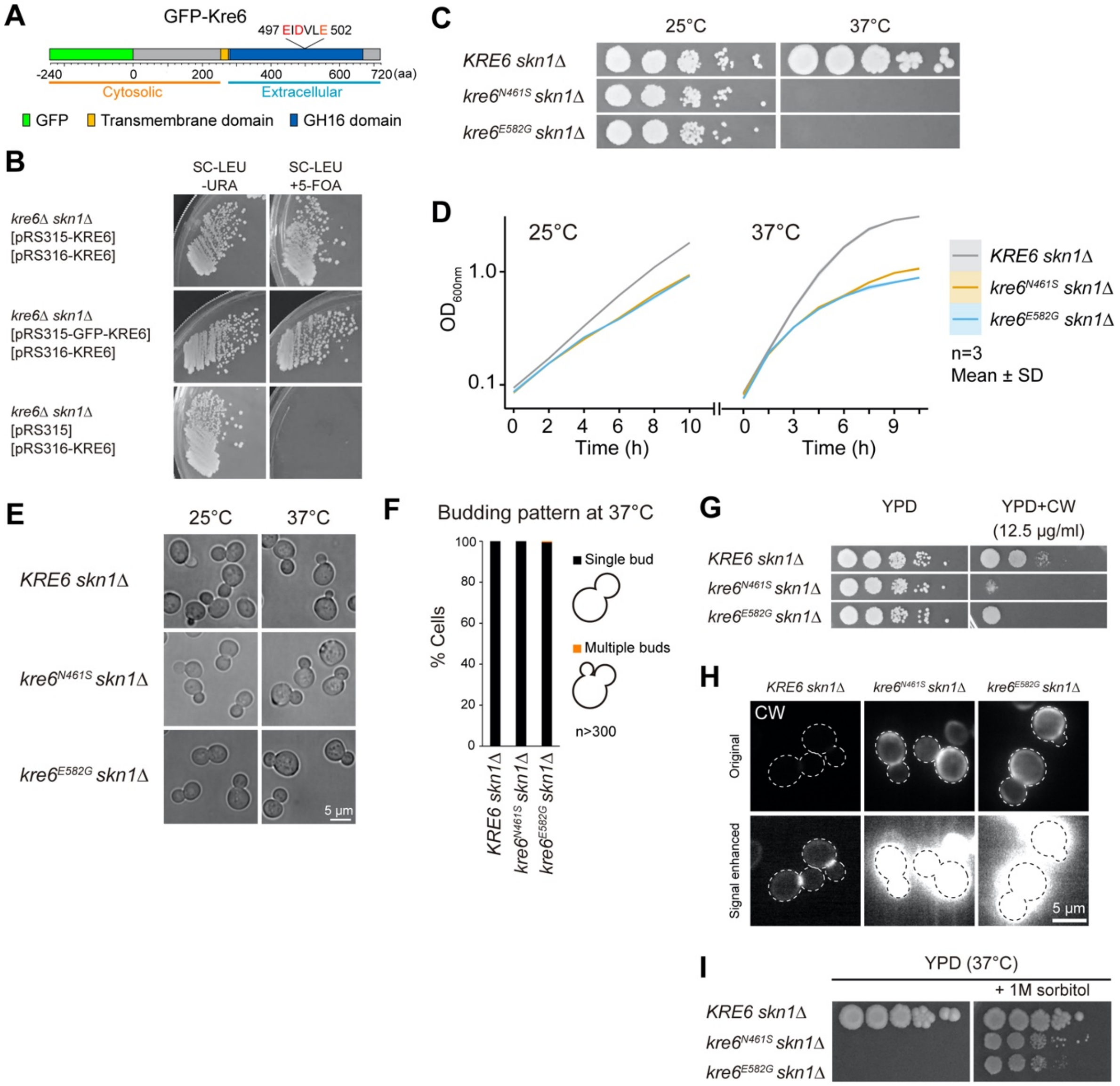

A
GFP-Kre6
497 EIDVLE 502
-240
0
200
400
600
720 (aa)
Cytosolic
Extracellular
GFP
Transmembrane domain
GH16 domain
B
SC-LEU -URA
SC-LEU +5-FOA
kre6Δ skn1Δ [pRS315-KRE6] [pRS316-KRE6]
kre6Δ skn1Δ [pRS315-GFP-KRE6] [pRS316-KRE6]
kre6Δ skn1Δ [pRS315] [pRS316-KRE6]
C
25°C
37°C
KRE6 skn1Δ
kre6N461S skn1Δ
kre6E582G skn1Δ
D
25°C
37°C
OD600nm
1.0
0.1
Time (h)
KRE6 skn1Δ
kre6N461S skn1Δ
kre6E582G skn1Δ
n=3
Mean ± SD
E
25°C
37°C
KRE6 skn1Δ
kre6N461S skn1Δ
kre6E582G skn1Δ
5 µm
F
Budding pattern at 37°C
% Cells
Single bud
Multiple buds
n>300
KRE6 skn1Δ
kre6N461S skn1Δ
kre6E582G skn1Δ
G
YPD
YPD+CW (12.5 µg/ml)
KRE6 skn1Δ
kre6N461S skn1Δ
kre6E582G skn1Δ
H
KRE6 skn1Δ
kre6N461S skn1Δ
kre6E582G skn1Δ
CW
Original
Signal enhanced
5 µm
I
YPD (37°C)
+ 1M sorbitol
KRE6 skn1Δ
kre6N461S skn1Δ
kre6E582G skn1Δ

Figure S5. Functional analysis of *GFP-KRE6* and *kre6-ts* mutants. Related to Figure 6.

(A) Diagram of GFP-Kre6. N-terminally tagged GFP is exposed to the cytosol. See also **Figure 6A**.

(B) The functionality of GFP-Kre6 was tested by examining the growth of cells carrying *LEU2*-plasmid of WT *KRE6* (pRS315-KRE6), *GFP-KRE6* (pRS315-GFP-KRE6), and empty vector (pRS315), on plates selecting for (SC-LEU-URA) or against (SC-LEU+5-FOA) the cover plasmid (pRS316-KRE6). Strains used are as follows: YEF10140 (*kre6Δ skn1Δ* [CEN, *URA3*, *KRE6*] [CEN, *LEU2*, *KRE6*]), YEF10141, and YEF10143. Cells were incubated at 25°C for four days. See also **Figure 6C**.

(C and D) The temperature sensitivity of the *kre6-ts* mutants was examined by testing colony formation on plates (C) or measuring optical density of liquid cell cultures (D). See Methods for detailed procedures. Cells were incubated at 25°C for four days in (C). Strains used are as follows: YOC5439 (*KRE6 skn1*Δ), YOC5428 (*kre6*^{N461S} *skn1*Δ), and YOC5376 (*kre6*^{E582G} *skn1*Δ).

(E and F) Morphology of *kre6-ts* cells at 25°C and 37°C. Cells were cultured to exponential phase at 25°C in YPD medium and split into two and diluted with fresh YPD medium to the 0.1 OD_{600}. Split cultures were incubated at 25°C or 37°C for 10 hours. Cells were then harvested, sonicated at 15% power for 15 seconds to declump, and imaged without fixation. Over 300 cells from (E) were counted for the budding pattern (F). Strains used are as follows: YOC5439, YOC5428, and YOC5376.

(G) The sensitivity of *kre6-ts* cells to CW was tested on YPD plates containing CW. Cells were incubated at 25°C for three days. Strains used are as follows: YOC5439, YOC5428, and YOC5376.

(H) Chitin deposition in the cell wall of *kre6-ts* cells. Cells used in (E and F) were resuspended in SC medium containing CW (10 μg/ml) to stain chitin in the cell wall.

(I) Suppression of temperature sensitivity of *kre6-ts* cells by osmotic support was determined by examining colony formation on YPD plates containing 1M sorbitol. Cells were incubated at 25°C for six days. Strains used are as follows: YOC5439, YOC5428, and YOC5376.

SUPPLEMENTAL TABLES

Table S1. Strains used in this study. Related to All Figures and TRANSPERANT METHODS.

Strain	Genotype	Source
YEF473A	*MAT*a *trp1-Δ63 leu2-Δ1 ura3-52 his3-Δ200 lys2-801*	(Bi and Pringle, 1996)
YEF473B	*MAT*α *trp1-Δ63 leu2-Δ1 ura3-52 his3-Δ200 lys2-801*	(Bi and Pringle, 1996)
BY4741	*MAT*a *his3Δ1 leu2Δ0 met15Δ0 ura3Δ0*	(Brachmann, et al., 1998)
BY4742	*MAT*α *his3Δ1 leu2Δ0 lys2Δ0 ura3Δ0*	(Brachmann, et al., 1998)
NCYC232	*Saccharomyces cerevisiae* Meyen ex E.C. Hansen (ATCC60782)	(Kurita, et al., 2011)
MOY882	As YEF473A except $cyk3^{TGc\Delta}$	(Onishi, et al., 2013)
YO1194	As YEF473B except *chs2Δ::His3MX6* [2μ, *URA3, CHS2*]	(Oh, et al., 2012)
YEF5804	As YEF473A except *CDC3::CDC3-mCherry-LEU2*	(Wloka, et al., 2013)
YEF7488	As YEF473A except *chs1Δ::KanMX6 chs3Δ::His3MX6*	Lab stock
YOC5376	As BY4742 except *kre6Δ::kanmx4::*$kre6^{E582G}$*-URA3 skn1Δ::KanMX4 met15Δ0*	This study[a]
YOC5428	As BY4741 except *kre6Δ::kanmx4::*$kre6^{N461S}$*-URA3 skn1Δ::KanMX4 lys2Δ0*	This study[a]
YOC5439	As BY4742 except *kre6Δ::kanmx4::KRE6-URA3 skn1Δ::KanMX4 met15Δ0*	This study[a]
YEF8174	As YEF473A except *fks1::URA3-kanMX6*	This study[b]
YEF8228	As YEF473A except GFP^{Envy}*-FKS1*	This study[c]
YEF8353	As YEF473A except *kre6::URA3-kanMX6*	This study[d]
YEF8361	As BY4741 except *fks1::URA3-kanMX6*	This study[e]
YEF8378	As YEF473A except GFP^{Envy}*-KRE6*	This study[f]
YEF8390	As YEF473A except *TUB1::HPH-pHIS3-mRuby2-TUB1*	(Marquardt, et al., 2020)

YEF8391	As YEF473A except *TUB1::HPH-pHIS3-mRuby2-TUB1 MLC2-mApple-URA3MX*	This study
YEF8392	As YEF473A except *TUB1::HPH-pHIS3-mRuby2-TUB1 MLC2-mApple-KanMX6*	This study
YEF8407	As BY4741 except *GFP^{Envy}-FKS1*	This study[g]
YEF8427	As YEF473A except *TUB1::HPH-pHIS3-mRuby2-TUB1 MLC2-mApple-URA3MX MOB2-3GFP-KanMX6*	This study
YEF8428	As YEF473A except *TUB1::HPH-pHIS3-mRuby2-TUB1 MLC2-mApple-URA3MX MYO2-GFP-TRP1*	This study
YEF8432	As YEF473A except *TUB1::HPH-pHIS3-mRuby2-TUB1 MLC2-mApple-URA3MX exo84::EXO84-GFP-TRP1*	This study[h]
YEF8434	As YEF473A except *TUB1::HPH-pHIS3-mRuby2-TUB1 MLC2-mApple-URA3MX CDC3::CDC3-GFP-LEU2*	This study[i]
YEF8435	As YEF473A except *TUB1::HPH-pHIS3-mRuby2-TUB1 MLC2-mApple-URA3MX fks1(1-789)::GFP^{Envy}-FKS1-LYS2*	This study[j]
YEF8533	As YEF473A except *TUB1::HPH-pHIS3-mRuby2-TUB1 MLC2-mApple-URA3MX MOB1-mNeonGreen-KanMX6*	This study
YEF8535	As YEF473A except *TUB1::HPH-pHIS3-mRuby2-TUB1 MLC2-mApple-URA3MX BNI1-GFP-KanMX6*	This study
YEF8625	As YEF473A except *TUB1::HPH-pHIS3-mRuby2-TUB1 MLC2-mApple-URA3MX kre6(1-341)::GFP^{Envy}-KRE6-LEU2*	This study[k]
YEF8626	As YEF473A except *TUB1::HPH-pHIS3-mRuby2-TUB1 MLC2-mApple-URA3MX MLC1::GFP-MLC1-LEU2*	This study[l]
YEF8627	As YEF473A except *TUB1::HPH-pHIS3-mRuby2-TUB1 MLC2-mApple-URA3MX HOF1-GFP-KanMX6*	This study
YEF8628	As YEF473A except *TUB1::HPH-pHIS3-mRuby2-TUB1 MLC2-mApple-URA3MX INN1-GFP-TRP1*	This study
YEF8631	As YEF473A except *CDC3::CDC3-mCherry-LEU2 HOF1-GFP-His3MX6*	This study
YEF9186	As YEF473A except *TUB1::HPH-pHIS3-mRuby2-TUB1 MLC2-mApple-URA3MX CTS1-GFP^{Envy}-KanMX6*	This study
YEF9189	As YEF473A except *TUB1::HPH-pHIS3-mRuby2-TUB1 MLC2-mApple-KanMX6 chs4(Δ1828-2091)::GFP-CHS4-URA3*	This study[m]
YEF9197	As YEF473A except *TUB1::HPH-pHIS3-mRuby2-TUB1 MLC2-mApple-URA3MX CYK3-GFP-His3MX6*	This study
YEF9198	As YEF473A except *TUB1::HPH-pHIS3-mRuby2-TUB1 MLC2-mApple-URA3MX ABP1-3GFP-KanMX6*	This study
YEF9200	As YEF473A except *TUB1::HPH-pHIS3-mRuby2-TUB1 MLC2-mApple-URA3MX BNR1-GFP-NatMX*	This study

YEF9242	As YEF473A except *TUB1::HPH-pHIS3-mRuby2-TUB1 MLC2-mApple-URA3MX fks1(1-789)::GFP*^{Envy}*-FKS1-LYS2 cyk3Δ::His3MX6*	This study
YEF9276	As YEF473A except *TUB1::HPH-pHIS3-mRuby2-TUB1 MLC2-mApple-URA3MX ACE2-3GFP-His3MX6*	This study
YEF9300	As YEF473A except *chs1Δ::KanMX6 chs3Δ::His3MX6 TUB1::HPH-pHIS3-mRuby2-TUB1*	This study[n]
YEF9498	As YEF473A except *TUB1::HPH-pHIS3-mRuby2-TUB1 MLC2-mApple-URA3MX leu2::pHIS3-GFP-TPM1-LEU2*	This study[o]
YEF9583	As YEF473A except *TUB1::HPH-pHIS3-mRuby2-TUB1 MLC2-mApple-URA3MX leu2::pHIS3-GFP-TPM1-LEU2 tpm1Δ::TRP1*	This study
YEF9592	As YEF473A except *chs1Δ::NatMX chs3Δ::His3MX6 TUB1::HPH-pHIS3-mRuby2-TUB1*	This study[p]
YEF9609	As YEF473A except *TUB1::HPH-pHIS3-mRuby2-TUB1 MLC2-mApple-URA3MX MYO1-GFP-His3MX6*	This study
YEF9610	As YEF473A except *TUB1::HPH-pHIS3-mRuby2-TUB1 MLC2-mApple-URA3MX IQG1-GFP-His3MX6*	This study
YEF9611	As YEF473A except *TUB1::HPH-pHIS3-mRuby2-TUB1 MLC2-mApple-URA3MX CHS2-GFP*^{Envy}*-His3MX6*	This study
YEF9612	As YEF473A except *TUB1::HPH-pHIS3-mRuby2-TUB1 MLC2-mApple-URA3MX CHS3-GFP-His3MX6*	This study
YEF9729	As YEF473A except *TUB1::HPH-pHIS3-mRuby2-TUB1 MLC2-mApple-URA3MX MYO1-GFP-His3MX6 cyk3Δ::NatMX*	This study
YEF9732	As YEF473A except *TUB1::HPH-pHIS3-mRuby2-TUB1 MLC2-mApple-URA3MX CHS2-GFP*^{Envy}*-His3MX6 cyk3Δ::NatMX*	This study
YEF9733	As YEF473A except *TUB1::HPH-pHIS3-mRuby2-TUB1 MLC2-mApple-URA3MX CHS3-GFP-His3MX6 cyk3Δ::NatMX*	This study
YEF9768	As YEF473A except *kre6Δ::KanMX4*	This study
YEF9787	As YEF473A except *cyk3*^{TGcΔ} *TUB1::HPH-pHIS3-mRuby2-TUB1*	This study[n]
YEF9803	As YEF473A except *crh1::URA3-kanMX6*	This study[q]
YEF9819	As YEF473A except *TUB1::HPH-pHIS3-mRuby2-TUB1 MLC2-mApple-URA3MX CDC14-GFP-His3MX6*	This study
YEF9820	As YEF473A except *cyk3*^{TGcΔ} *TUB1::HPH-pHIS3-mRuby2-TUB1 MLC2-mApple-URA3MX*	This study
YEF9833	As YEF473A except *cyk3*^{TGcΔ} *TUB1::HPH-pHIS3-mRuby2-TUB1 MLC2-mApple-URA3MX CHS2-GFP*^{Envy}*-His3MX6*	This study

YEF9837	As YEF473A except *CRH1-GFP*^{Envy}	This study[r]
YEF9847	As YEF473A except *cyk3*$^{TGc\Delta}$*-GFP-His3MX TUB1::HPH-pHIS3-mRuby2-TUB1 MLC2-mApple-URA3MX*	This study
YEF9848	As YEF473A except *CRH1-GFP*^{Envy} *MLC2-mApple-URA3MX*	This study
YEF9863	As YEF473A except *CRH1-GFP*^{Envy} *MLC2-mApple-URA3MX TUB1::HPH-pHIS3-mRuby2-TUB1*	This study[n]
YEF9871	As YEF473A except *kre6Δ::NatMX*	This study[p]
YEF9894	As YEF473B except *chs2Δ::His3MX6 TUB1::HPH-pHIS3-mRuby2-TUB1*[2μ, *URA3, CHS2*]	This study[n]
YEF9900	As YEF473A except *kre6Δ::NatMX* [CEN, *URA3*, *KRE6*]	This study[s]
YEF9905	As YEF473B except *chs2Δ::His3MX6 TUB1::HPH-pHIS3-mRuby2-TUB1 MLC2-mApple-KanMX6* [2μ, *URA3, CHS2*]	This study
YEF9911	As YEF473A except *kre6Δ::NatMX skn1Δ::TRP1* [CEN, *URA3, KRE6*]	This study
YEF9921	As YEF473B except *chs2Δ::His3MX6 TUB1::HPH-pHIS3-mRuby2-TUB1 MLC2-mApple-KanMX6 leu2::chs2*^{I750A}*-GFP-KanMX6-LEU2* [2μ, *URA3, CHS2*]	This study[t]
YEF10092	As YEF473B except *chs2Δ::His3MX6 TUB1::HPH-pHIS3-mRuby2-TUB1 MLC2-mApple-KanMX6 leu2::chs2*^{S751A}*-GFP-KanMX6-LEU2* [2μ, *URA3, CHS2*]	This study[t]
YEF10093	As YEF473B except *chs2Δ::His3MX6 TUB1::HPH-pHIS3-mRuby2-TUB1 MLC2-mApple-KanMX6 leu2::chs2*^{N797Q}*-GFP-KanMX6-LEU2* [2μ, *URA3, CHS2*]	This study[t]
YEF10098	As YEF473B except *chs2Δ::His3MX6 TUB1::HPH-pHIS3-mRuby2-TUB1 MLC2-mApple-KanMX6 leu2::CHS2-GFP-KanMX6-LEU2*	This study[t]
YEF10100	As YEF473B except *chs2Δ::His3MX6 TUB1::HPH-pHIS3-mRuby2-TUB1 MLC2-mApple-KanMX6 leu2::chs2*^{S751A}*-GFP-KanMX6-LEU2*	This study[t]
YEF10101	As YEF473B except *chs2Δ::His3MX6 TUB1::HPH-pHIS3-mRuby2-TUB1 MLC2-mApple-KanMX6 leu2::chs2*^{N797Q}*-GFP-KanMX6-LEU2*	This study[t]
YEF10128	As YEF473B except *chs2Δ::His3MX6 TUB1::HPH-pHIS3-mRuby2-TUB1 MLC2-mApple-KanMX6 leu2::chs2*^{N797Q}*-GFP-KanMX6-LEU2* [2μ, *TRP1, CYK3*]	This study[u]

YEF10129	As YEF473B except *chs2Δ::His3MX6 TUB1::HPH-pHIS3-mRuby2-TUB1 MLC2-mApple-KanMX6 leu2::chs2*^{N797Q}*-GFP-KanMX6-LEU2* [2μ, *TRP1*]	This study[u]
YEF10140	As YEF473A except *kre6Δ::NatMX skn1Δ::TRP1* [CEN, *URA3*, *KRE6*] [CEN, *LEU2*, *KRE6*]	This study[v]
YEF10141	As YEF473A except *kre6Δ::NatMX skn1Δ::TRP1* [CEN, *URA3*, *KRE6*] [CEN, *LEU2*, *GFP*^{Envy}*-KRE6*]	This study[v]
YEF10142	As YEF473A except *kre6Δ::NatMX skn1Δ::TRP1* [CEN, *URA3*, *KRE6*] [CEN, *LEU2*, *GFP*^{Envy}*-kre6*^{QNQ}]	This study[v]
YEF10143	As YEF473A except *kre6Δ::NatMX skn1Δ::TRP1* [CEN, *URA3*, *KRE6*] [CEN, *LEU2*]	This study[v]
YEF10153	As YEF473B except *chs2Δ::His3MX6 TUB1::HPH-pHIS3-mRuby2-TUB1 MLC2-mApple-KanMX6 leu2::CHS2-GFP-KanMX6-LEU2 cyk3Δ::NatMX*	This study
YEF10246	As YEF473A except *chs1Δ::NatMX chs3Δ::His3MX6 TUB1::HPH-pHIS3-mRuby2-TUB1 MLC2-mApple-URA3MX*	This study
YEF10247	As YEF473A except *TUB1::HPH-pHIS3-mRuby2-TUB1 MLC2-mApple-URA3MX kre6Δ::KanMX4*	This study
YEF10248	As YEF473A *kre6Δ::KanMX4 leu2::KRE6-LEU2*	This study[w]
YEF10249	As YEF473A *kre6Δ::KanMX4 leu2::LEU2*	This study[w]
YEF10250	As YEF473A *kre6Δ::KanMX4 leu2::kre6*^{QNQ}*-LEU2*	This study[w]
YEF10290	As YEF473A except *TUB1::HPH-pHIS3-mRuby2-TUB1 MLC2-mApple-URA3MX fks1(1-789)::GFP*^{Envy}*-FKS1-LYS2 lrg1Δ::KanMX4*	This study
YEF10291	As YEF473A except *TUB1::HPH-pHIS3-mRuby2-TUB1 MLC2-mApple-URA3MX CHS2-GFP*^{Envy}*-His3MX6 lrg1Δ::KanMX4*	This study
YEF10292	As YEF473A except *TUB1::HPH-pHIS3-mRuby2-TUB1 MLC2-mApple-URA3MX CHS3-GFP-His3MX6 lrg1Δ::KanMX4*	This study
YEF10337	As YEF473A except *TUB1::HPH-pHIS3-mRuby2-TUB1 MLC2-mApple-URA3MX kre6Δ::KanMX4::GFP*^{ENVY}*-kre6*^{QNQ}*-LEU2*	This study[x]
YEF10361	As YEF473B except *chs2Δ::HIS3 TUB1::HPH-pHIS3-mRuby2-TUB1 MLC2-mApple-KanMX6 leu2::CHS2-GFP-KanMX6-LEU2* [2μ, *URA3*, *CHS2*]	This study[t]
YEF10368	As YEF473A except *chs1Δ::NatMX chs3Δ::His3MX6 TUB1::HPH-pHIS3-mRuby2-TUB1 MLC2-mApple-URA3MX CHS2-GFP-TRP1*	This study

YEF10369	As YEF473A except *chs1Δ::NatMX chs3Δ::His3MX6 TUB1::HPH-pHIS3-mRuby2-TUB1 MLC2-mApple-URA3MX* [2μ, *TRP1*, *CYK3*]	This study[y]
YEF10370	As YEF473A except *chs1Δ::NatMX chs3Δ::His3MX6 TUB1::HPH-pHIS3-mRuby2-TUB1 MLC2-mApple-URA3MX* [2μ, *TRP1*, $cyk3^{TGc\Delta}$]	This study[y]
YEF10371	As YEF473A except *chs1Δ::NatMX chs3Δ::His3MX6 TUB1::HPH-pHIS3-mRuby2-TUB1 MLC2-mApple-URA3MX* [2μ, *TRP1*]	This study[y]
YEF10378	As YEF473B except *chs2Δ::His3MX6 TUB1::HPH-pHIS3-mRuby2-TUB1 MLC2-mApple-KanMX6 leu2::*$chs2^{N797Q}$*-GFP-KanMX6-LEU2* [2μ, *TRP1*, $cyk3^{TGc\Delta}$]	This study[u]
YEF10443	As YEF473B except *chs2Δ::His3MX6 TUB1::HPH-pHIS3-mRuby2-TUB1 MLC2-mApple-KanMX6 leu2::*$chs2^{I750A}$*-GFP-KanMX6-LEU2*	This study[t]

Except where noted, gene deletion or tagging at the chromosomal locus was constructed using a PCR method as described previously (Slubowski, et al., 2015; Lee, et al., 2013; Longtine, et al., 1998). Primers used for the PCR are listed in **Table S2**.

[a] See Transparent Methods.

[b] A DNA fragment (*URA3-KanMX6* cassette) was amplified by PCR using the plasmid pFA6a-URA3-KanMX6 as the template DNA and the pair of primers with 40-bp flanking sequence of the *FKS1* start codon (P217 and P498), and the DNA fragment was then inserted at the *FKS1* locus in YEF473A to replace the start codon.

[c] A DNA fragment (GFP^{Envy}) was amplified by PCR using the plasmid pFA6a-GFPEnvy-KanMX6 as the template DNA and the pair of primers with 40-bp flanking sequence of the *FKS1* start codon (P217 and P506), and the DNA fragment was then used to replace the *URA3-KanMX6* cassette in YEF8174.

[d] A DNA fragment (*URA3-KanMX6* cassette) was amplified by PCR using the plasmid pFA6a-URA3-KanMX6 as the template DNA and the pair of primers with 40-bp flanking sequence of the *KRE6* start codon (P215 and P496), and the DNA fragment was then inserted at the *KRE6* locus of YEF473A to replace the start codon.

[e] A DNA fragment (*fks1::URA3-KanMX6* cassette) was amplified by PCR using the chromosomal DNA of YEF8174 as the template DNA and the pair of primers (P97 and P498), and the DNA fragment was then inserted at the *FKS1* locus in BY4741.

[f] A DNA fragment (*GFP^{Envy}*) was amplified by PCR using the plasmid pFA6a-GFPEnvy-KanMX6 as the template DNA and the pair of primers with 40-bp flanking sequence of the *KRE6* start codon (P215 and P504), and the DNA fragment was then used to replace the *URA3-KanMX6* cassette in YEF8353.

[g] A DNA fragment (*GFP^{Envy}*) was amplified by PCR using the chromosomal DNA of YEF8228 as the template DNA and the pair of primers (P96 and P506), and the DNA fragment was then used to replace the *URA3-KanMX6* cassette in YEF8361.

[h] BglII-digested plasmid pG366 (a gift from Wei Guo) was integrated into *EXO84* locus. As a result, the endogenous copy is disrupted.

[i] BglII-digested plasmid YIp128-CDC3-mCherry was integrated into the *CDC3* locus.

[j] HindIII-digested plasmid pRS307-ENVY-FKS1(1-789) was integrated into the *FKS1* locus. As a result, the endogenous copy is truncated.

[k] NheI-digested plasmid pRS305-ENVY-KRE6(1-341) was integrated into the *KRE6* locus. As a result, the endogenous copy is truncated.

[l] Tth111I-digested plasmid YIplac128-GFP-MLC1 was integrated into the *MLC1* locus.

[m] NheI-digested plasmid pJL68 (a gift from Kelly Tatchell) was integrated into the *CHS4* locus. As a result, the endogenous copy is truncated.

[n] XbaI-digested plasmid pHis3:mRuby2-Tub1+3'UTR::HPH was integrated into the *TUB1* locus.

[o] EcoRV-digested plasmid YIp128-proHIS3-yEGFP-TPM1-tADH1 was integrated into the *LEU2* locus.

[p] A DNA fragment (*NatMX* cassette) from EcoRI-digested plasmid p4339 was used to switch the *KanMX4* cassette at a chromosomal locus.

[q] A DNA fragment (*URA3-KanMX6* cassette) was amplified by PCR using the plasmid pFA6a-URA3-KanMX6 (a gift from John Pringle) as the template DNA and the pair of primers with 40-bp flanking sequence of the site between codons 56 and 57 in the *CRH1* ORF (P300 and P589), and the DNA fragment was then inserted at the *CRH1* locus of YEF473A.

[r] A DNA fragment (*GFP^{Envy}*) was amplified by PCR using the plasmid pFA6a-GFPEnvy-KanMX6 as the template DNA and the pair of primers with 40-bp flanking sequence of the site between codons 56 and 57 in the *CRH1* ORF (P300 and P590), and the DNA fragment was then used to replace the *URA3-KanMX6* cassette in YEF9803.

[s] Transformed with low-copy plasmid pYO3165 (pRS316-KRE6).

[t] AflII-digested plasmid pRS305-CHS2-GFP, pRS305-CHS2(I750A)-GFP, pRS305-CHS2(S751A)-GFP, or pRS305-CHS2(N797Q)-GFP was integrated into the *LEU2* locus of YEF9905 to generate YEF10361, YEF9921, YEF10092, or YEF10093, respectively. These strains were further selected on SC+5-FOA plate to remove the *CHS2* cover plasmid to generate YEF10098, YEF10443, YEF10100, or YEF10101, respectively.

[u] Multi-copy plasmid pBK42 (a gift from John Chant), YEplac112-CYK3(TGcΔ), or YEplac112 was transformed into YEF10093, and the Trp+ transformants were then selected on SC-Trp+5-FOA plate to remove the *CHS2* cover plasmid to generate YEF10128, YEF10378, or YEF10129, respectively.

[v] Low-copy plasmid pRS315-KRE6, pRS315-ENVY-KRE6, pRS315-ENVY-KRE6QNQ, or pRS315 was transformed into YEF9911 to generate YEF10140, YEF10141, YEF10142, or YEF10143, respectively.

[w] BstEII-digested plasmid pRS305-KRE6, pRS305-KRE6QNQ, or pRS305 was integrated into the *LEU2* locus of YEF9768 to generate YEF10248, YEF10250, or YEF10249, respectively.

[x] BspEI-digested plasmid pRS305-ENVY-KRE6QNQ was integrated into the promoter region of *KRE6* locus of YEF10247 to generate YEF10337.

[y] Multi-copy plasmid pBK42, YEplac112-CYK3(TGcΔ), or YEplac112 was transformed into YEF10246 to generate YEF10369, YEF10370, or YEF10371, respectively.

Table S2. Oligonucleotides and plasmids used in this study. Related to All Figures and TRANSPARENT METHODS.

Oligonucleotides	Function	Identifier
F219-FKS1-40up-from-start-F1: CAAGTAGCTGAAATCAAGTCTTTCATACAACGGTCAGACCCGGATCCCCGGGTTAATTAA	For the construction of *fks1::URA3-KanMX6* and *GFP*^{Envy}*-FKS1*	P217
R181-FKS1-40down-from-start-R1: TATAGTCCGTTTGGCCCTGATAAGGTTGTTGATCAGTGTTGAATTCGAGCTCGTTTAAAC	For the construction and amplification of *fks1::URA3-KanMX6*	P498
R189-FKS1-40down-from-start-ENVY: TATAGTCCGTTTGGCCCTGATAAGGTTGTTGATCAGTGTTTTTGTACAATTCGTCCATTC	For the construction and amplification of *GFP*^{ENVY}*-FKS*1	P506
F217-KRE6-40up-from-start-F1: AAGTTTACAGAATAGGTATACCACTCACTCCTTTACTCTACGGATCCCCGGGTTAATTAA	For the construction of *kre6::URA3-KanMX6* and *GFP*^{Envy}*-KRE6*	P215
R179-KRE6-40down-from-start-R1: TGCTACTAAAGTTGTGCGTTTCAGTTAGATTTCTCAAAGGGAATTCGAGCTCGTTTAAAC	For the construction of *kre6::URA3-KanMX6*	P496
F099-FKS1-seq02: TACAGCTGTTTTAACCGA	For the amplification of *fks1::URA3-KanMX6*	P97
R187-KRE6-40down-from-start-ENVY: TGCTACTAAAGTTGTGCGTTTCAGTTAGATTTCTCAAAGGTTTGTACAATTCGTCCATTC	For the construction of *GFP*^{Envy}*-KRE6*	P504
F098-FKS1-seq01: GCAAAAAGAGAAAACCAA	For the amplification of *GFP*^{Envy}*-FKS*1	P96
Mlc2-Amp+300-Rev: TCCATTGCGTATTGCCATG	For the amplification of *MLC2-mApple-URA3MX* and *MLC2-mApple-KanMX6*	Y316
Mlc2-Amp-350-For: GGACACGTAATGATTAGTAG	For the amplification of *MLC2-mApple-URA3MX* and *MLC2-mApple-KanMX6*	Y317
F235-MOB2-310up-from-Stop: TGGCCCTCACGTGGATAAATAAC	For the amplification of *MOB2-3GFP-KanMX6*	P233
R210-MOB2-330down-from-Stop: ACATCACTGACTTGGACGCACTTG	For the amplification of *MOB2-3GFP-KanMX6*	P527
F237-MYO2-300up-from-Stop: TTGATTTCACAATACCAGGTGGCAG	For the amplification of *MYO2-GFP-TRP1*	P235
R212-MYO2-300down-from-Stop: GGTAATTATCGTTCCTATCGTCGT	For the amplification of *MYO2-GFP-TRP1*	P529

F234-MOB1-300up-from-Stop: TAAGTATGTGGAATGTTTGATGAGGTG	For the amplification of *MOB1-mNeonGreen-KanMX6*	P232
R209-MOB1-tag-R3: GAAGAATACAACCTACAAGCAGACTTATATAAATATACAATATCGATGAATTCGAGCTCG	For the amplification of *MOB1-mNeonGreen-KanMX6*	P526
F236-BNI1-310up-from-Stop: TCTATTCTTTATTCTCCAGAAGCTATG	For the amplification of *BNI1-GFP-KanMX6*	P234
R211-BNI1-300down-from-Stop: CTGTGCTTGTCACTTAGGTAAACGAG	For the amplification of *BNI1-GFP-KanMX6*	P528
F181-HOF1-300up-from-Stop: ACAGGAAACATCATGGATGAGAGAG	For the amplification of *HOF1-GFP-KanMX6*	P179
R145-HOF1-300down-from-Stop: GTCATTAGAGCACATTAAATAATATG	For the amplification of *HOF1-GFP-KanMX6*	P462
F003-INN1-INT1: CAATACGCCGTTAGTAAGAA	For the amplification of *INN1-GFP-TRP1*	P3
R002-INN1-3'-500: AAACATCGGACTATCCATGCT	For the amplification of *INN1-GFP-TRP1*	P320
F180-HOF1-tag-F5: TTAATTCCCTATAATTTCATTCAGCTACTGCATCAAGGTCTTGGTGACGGTGCTGGTTTA	For the amplification of *HOF1-GFP-His3MX6*	P178
R144-HOF1-tag-R3: TTTCTTTTATCAGAAAACTAGTAAAATTGATATACATCGAGATCGATGAATTCGAGCTCG	For the amplification of *HOF1-GFP-His3MX6*	P461
Cts1-ORF-F: TGACGAGTAGCACCAAGA	For the amplification of *CTS1-GFP^{Envy}-KanMX6*	N/A
Cts1-Flank-R: TTTCACCGCATAGACCAA	For the amplification of *CTS1-GFP^{Envy}-KanMX6*	N/A
F204-CYK3-300up-from-Stop: ATTGTCGAGCCACAGAGTAGATATC	For the amplification of *CYK3-GFP-His3MX6*	P202
R168-CYK3-300down-from-Stop: GAAGTAGCACTCAATTGGTTTTCAGG	For the amplification of *CYK3-GFP-His3MX6*	P485
F118-ABP1-250up-from-Stop: CCATCAAGAAGCTCTGCAGCTCC	For the amplification of *ABP1-3GFP-KanMX6*	P116
R089-INN1-K31A-mutagenesis: CCTAAGCATTACATTTTGAGCGTCCAGTTTATTCAAATTAG	For the amplification of *ABP1-3GFP-KanMX6*	P406

F247-BNR1-300up-from-Stop: AGAGTGTACGAACAAAGGAAGAGC	For the amplification of *BNR1-GFP-NatMX*	P241
R211-BNI1-300down-from-Stop: CTGTGCTTGTCACTTAGGTAAACGAG	For the amplification of *BNR1-GFP-NatMX*	P528
CYK3-F-Check: AAGGCCAAAATTAACGGGAC	For the amplification of *cyk3*Δ::*His3MX6* and *cyk3*Δ*::NatMX*	N/A
R168-CYK3-300down-from-Stop: GAAGTAGCACTCAATTGGTTTTCAGG	For the amplification of *cyk3*Δ::*His3MX6* and *cyk3*Δ*::NatMX*	P485
Ace2-Check-F: CTGTCTCAGAATGTCCCAATA	For the amplification of *ACE2-3GFP-His3MX6*	N/A
Ace2-Check-R: GGTCTAACCAACAGGGTGTAT	For the amplification of *ACE2-3GFP-His3MX6*	N/A
F297-TPM1-40up-from-start-F1: ATAGTAGAACTCACACCCCATACACACAAAAAAGGCAACACGGATCCCCGGGTTAATTAA	For the construction of *tpm1*Δ*::TRP1*	P290
R270-TPM1-40down-from-stop-R1: GATTTGCTCAAGAACAAGGAACAAAGACCAGCGTGTTGGGGAATTCGAGCTCGTTTAAAC	For the construction of *tpm1*Δ*::TRP1*	P581
Myo1-159bp-US-Stop: CTAGCGAATAAAAATAGAAGCGA	For the amplification of *MYO1-GFP-His3MX6*	N/A
Myo1-249bp-DS-Stop: GATACGGGGTGAAAGAGTT	For the amplification of *MYO1-GFP-His3MX6*	N/A
F183-IQG1-300up-from-Stop: ACTAGCTGAACTGACTGTAAAAGTC	For the amplification of *IQG1-GFP-His3MX6*	P181
R147-IQG1-300down-from-Stop: AAATGATGATTGCCAACTATAGTAGCC	For the amplification of *IQG1-GFP-His3MX6*	P464
F123-CHS2-300up-from-Stop: GAATTGTGATGATTTGGATGC	For the amplification of *CHS2-GFP^{Envy}-KanMX6* and *CHS2-GFP^{Envy}-His3MX6*	P121
R093-CHS2-300down-from-Stop: TCAAAAGCTCTTGATGCCCA	For the amplification of *CHS2-GFP^{Envy}-KanMX6, CHS2-GFP^{Envy}-His3MX6* and *CHS2-GFP-TRP1*	P410
F077-CHS3-250up-from-Stop: TGCTACGAGATGGTCGTACCTATGGTGG	For the amplification of *CHS3-GFP-His3MX6*	P75
R062-CHS3-250down-from-Stop: GCCACAGATAAATAGTGGCATATTCGG	For the amplification of *CHS3-GFP-His3MX6*	P379
F152-KRE6-270up: AGCTTACAGAAGCACTTAAGAATC	For the amplification of *kre6*Δ::*kanMX4*	P150

R115-KRE6-250down: TGTATACCTATATAAACAATCTTAGAGG	For the amplification of *kre6*Δ::*kanMX4*	P432
F307-CRH1-40up-SpeI-F1: AAAAACTACAGGTTGTACGCCGGATACAGCTTTGGCAACTCGGATCCCCGGGTTAATTAA	For the construction of *crh1::URA3-KanMX6 and CRH1-GFP^{envy}*	P300
R278-CRH1-40down-SpeI-R1: TAAACCATTTGGATGAAGATGAGAAATCTTCGCTAAAACTGAATTCGAGCTCGTTTAAAC	For the construction of *crh1::URA3-KanMX6*	P589
F262-450up-CDC14: TGCCAGAAATTCGAGCCAACCTTCTACG	For the amplification of *CDC14-GFP-His3MX6*	P256
R033-CDC14-yoEGFP-tag: TGCATAAATCAGGCAAGTTACTATTGCCGGTATACATATGACTCGATGAATTCGAGCTCG	For the amplification of *CDC14-GFP-His3MX6*	P351
R279-CRH1-40down-SpeI-ENVY: TAAACCATTTGGATGAAGATGAGAAATCTTCGCTAAAACTTTTGTACAATTCGTCCATTC	For the construction of *CRH1-GFP^{envy}*	P590
F218-SKN1-40up-from-start-F1: TAACCATTACTAGCTAGTGTTACATAAACAATAGCAGAATCGGATCCCCGGGTTAATTAA	For the construction of *skn1*Δ*::TRP1*	P216
R287-SKN1-R1: ATTTTGCCTCCTTAGAATTAAAGCGCACTTAGATAGTAGGGAATTCGAGCTCGTTTAAAC	For the construction of *skn1*Δ*::TRP1*	P598
P641-F-600up-LRG1-from-start: ACCTCAAAACTGAAATTATGGGCAAAC	For the amplification of *lrg1*Δ::*kanMX4*	P641
P642-R-500down-LRG1-from-stop: TATTTAGAGAATAAGTTACTGGTGGG	For the amplification of *lrg1*Δ::*kanMX4*	P642
F145-CHS2-1000up-MCS: AGCTCCACCGCGGTGTCTTGCTGGATCACAAGTTATAAATAATG	For the amplification of *CHS2-GFP-TRP1*	P143
F312-CHS2(I750A)-mutagenesis: ACATCGTTGCCTCATTGCTCTCCAC	For the site-directed mutagenesis of *CHS2*	P305
F313-CHS2(S751A)-mutagenesis: TCGTTATCGCATTGCTCTCCACCTATG	For the site-directed mutagenesis of *CHS2*	P306
F314-CHS2(N797Q)-mutagenesis: CTTCTGTCAGACACACGACGTTTCCTG	For the site-directed mutagenesis of *CHS2*	P307

R284-CHS2(I750A)-mutagenesis: CAATGAGGCAACGATGTCGACAAATA	For the site-directed mutagenesis of *CHS2*	P595
R285-CHS2(S751A)-mutagenesis: AGCAATGCGATAACGATGTCGACAAAT	For the site-directed mutagenesis of *CHS2*	P596
R286-CHS2(N797Q)-mutagenesis: CGTGTGTCTGACAGAAGGCAAAAATCTG	For the site-directed mutagenesis of *CHS2*	P597
F231-pRS-MCS(SacI)-KRE6-1kb-up: CTATAGGGCGAATTGGAGCTCCGTCATCCTGAACGTTCATAAAACACATATGAAACAACC	For the construction of pRS315-KRE6, pRS315-ENVY-KRE6, and pRS305-ENVY-KRE6(1-341)	P229
R295-pRS-MCS(ApaI)-KRE6-term: CAAAAGCTGGGTACCGGGCCCAATTATTTACCTCATAGGGTACACAATATCCGTGAG	For the construction of pRS315-KRE6 and pRS315-ENVY-KRE6	P606
F324-KRE6-mutagenesis-E497Q-D499N-E502Q: CAAATTAATGTTTTGCAAGGTGAAACTGATACTAAGATTG	For the site-directed mutagenesis of pRS305-KRE6 or pRS315-ENVY-KRE6	P317
R294-KRE6-mutagenesis-E497Q-D499N-E502Q: TTGCAAAACATTAATTTGTGGAGCTCCTCTACCAACACCTTG	For the site-directed mutagenesis of pRS305-KRE6 or pRS315-ENVY-KRE6	P605
R205-pRS-MCS(ApaI)-KRE6-aa341-with-stop: CAAAAGCTGGGTACCGGGCCCCTAAAATGTTCTGCCTTCTGCATTAAACTCATCAGAAAA	For the construction of pRS305-ENVY-KRE6(1-341)	P522
F224-pRS-MCS(SacI)-FKS1-1kb-up: CTATAGGGCGAATTGGAGCTCGCAAAAAGAGAAAACCAAGGGACCAGAACAAAGCAAAAT	For the construction of pRS316-ENVY-FKS1(1-788) and pRS307-ENVY-FKS1(1-788)	P222
R195-pRS-MCS(ApaI)-FKS1-aa790-with-stop: CAAAAGCTGGGTACCGGGCCCCTAAGTTCTTTTACCTTCGATTTCAGATGGAACTTGATG	For the construction of pRS316-ENVY-FKS1(1-788) and pRS307-ENVY-FKS1(1-788)	P512
F295-linker(SpeI)-TPM1: TAGAACTAGTGGTGGTAGCGGAGGAACAGGTGGTATGGACAAAATCAGAGAAAAGCTAAG	For the construction of YIp128-proHIS3-yEGFP-TPM1-tADH1	P288
R269-TPM1-Stop-AscI: GAAGTGGCGCGCCTCACAAGTTTTCCAGAGATGCAGCAATTTCGTCCAGTTCCTT	For the construction of YIp128-proHIS3-yEGFP-TPM1-tADH1	P580

P632-R-CYK3-GTc-deletion: ATTGGGTTGGTTACATTTCCTTTCTCTAAATTTTGGGGTTCCGCAGAAAAATCTGACGTT	For the construction of YEplac112-CYK3$^{TGc\Delta}$	P632
P633-F-CYK3-GTc-deletion: AACCCCAAAATTTAGAGAAAGGAAATGTAACCAACCCAATTCATGAATTTGTAAATAACA	For the construction of YEplac112-CYK3$^{TGc\Delta}$	P633

**Plasmids**	**Source**	**Identifier**
p4339	(Tong, et al., 2001)	
pFA6a-3GFP-KanMX6	(Wu, et al., 2006)	
pFA6a-GFP(S65T)-kanMX6	(Longtine, et al., 1998)	
pFA6a-GFP(S65T)-TRP1	(Longtine, et al., 1998)	
pFA6a-GFPEnvy-KanMX6	This study	BiLab collection#E2341
pFA6a-His3MX6	(Longtine, et al., 1998)	
pFA6a-link-yomApple-CaURA3	(Lee, et al., 2013)	Addgene Cat#44879
pFA6a-link-yomApple-Kan	(Lee, et al., 2013)	Addgene Cat#44957
pFA6a-link-ymNeonGreen-KanMX6	This study	BiLab collection#E2265
pFA6a-link-yoEGFP-SpHIS5	(Lee, et al., 2013)	Addgene Cat#44836
pFA6a-link-GFPEnvy-KanMX6	This study	BiLab collection#E2282
pFA6a-link-GFPEnvy-SpHis5	(Slubowski, et al., 2015)	Addgene Cat#60782
pFA6a-link-yoEGFP-NatMX6	(Marquardt, et al., 2020)	BiLab collection#E2375
pFA6a-TRP1	(Longtine, et al., 1998)	
pG366	Wei Guo	
pHis3:mRuby2-Tub1+3'UTR::HPH (bWL715)	(Markus, et al., 2015)	
pRS305	(Sikorski and Hieter, 1989)	
pRS305-CHS2-GFP	(Wloka, et al., 2011)	BiLab collection#E2529

pRS305-CHS2(I750A)-GFP	This study	BiLab collection#E2531
pRS305-CHS2(S751A)-GFP	This study	BiLab collection#E2532
pRS305-CHS2(N797Q)-GFP	This study	BiLab collection#E2533
pRS305-KRE6	This study	BiLab collection#E2577
pRS305-KRE6^{QNQ}	This study	BiLab collection#E2580
pRS305-ENVY-KRE6(1-341)	This study	BiLab collection#E2380
pRS305-ENVY-KRE6^{QNQ}	This study	BiLab collection#E2578
pRS306-GFP-Chs4Δ610-696 (pJL68)	(Larson, et al., 2008)	
pRS307	(Eriksson, et al., 2004)	Addgene Cat#51785
pRS307-ENVY-FKS1(1-789)	This study	BiLab collection#E2361
pRS315	(Sikorski and Hieter, 1989)	
pRS315-KRE6	This study	BiLab collection#E2556
pRS315-ENVY-KRE6	This study	BiLab collection#E2557
pRS315-ENVY-KRE6^{QNQ}	This study	BiLab collection#E2558
pRS316	(Sikorski and Hieter, 1989)	
pRS316-ENVY-FKS1(1-789)	This study	BiLab collection#E2356
pRS316-KRE6 (pYO3165)	This study	
SB221	(Ben-Aroya, et al., 2008)	
YIplac128-GFP-MLC1	(Feng, et al., 2015)	BiLab collection#E2062
YIp128-CDC3-GFP	(Gao, et al., 2007)	BiLab collection#E1915
YIp128-proHIS3-yEGFP-TPM1-tADH1	This study	BiLab collection#E2497

YEplac112	(Gietz and Sugino, 1988)	
YEplac112-CYK3 (pBK42)	(Korinek, et al., 2000)	
YEplac112-CYK3$^{TGc\Delta}$	This study	BiLab collection#E2598

Table S3. Statistics of LatA effects on kinetics of cytokinetic proteins. Related to Figure 4.

			Maximum intensity					Recruitment rate (intensity/min)				Removal rate (intensity/min)			
			Intensity (a.u.)			Time point (min)									
Protein	Treatment	n	Mean	SD	% to DMSO	At Max.	From DMSO	Mean	SD	% to DMSO	Time point (min)	Mean	SD	% to DMSO	Time point (min)
Myo1	DMSO	21	6927	1084	–	-10.5	–	83	91	–	-16.5, -15, -13.5	-951	291	–	0, 1.5, 3
	LatA	22	6269	1406	91%	-10.5	0	69	176	83%	-16.5, -15, -13.5	-462	172	49%	0, 1.5, 3
Iqg1	DMSO	22	2848	633	–	-3	–	196	54	–	-15, -13.5, -12, -10.5	-644	231	–	-1.5, 0, 1.5
	LatA	20	2384	740	84%	-6	-3	116	54	59%	-16.5, -15, -13.5, -12	-161	58	25%	-1.5, 0, 1.5, 3
Mlc1	DMSO	22	13978	2323	–	-1.5	–	452	230	–	-16.5, -15, -13.5, -12, -10.5	-1549	507	–	0, 1.5, 3
	LatA	27	9235	1316	66%	-4.5	-3	384	122	85%	-16.5, -15, -13.5, -12, -10.5	-489	162	32%	0, 1.5, 3
Myo2	DMSO	20	9056	1934	–	7.5	–	596	220	–	-6, -4.5, -3, -1.5	-394	228	–	9, 10.5, 12, 13.5
	LatA	21	4772	918	53%	24	16.5	70	98	12%	-6, -4.5, -3, -1.5	-47	56	12%	31.5, 33, 34.5, 36, 37.5, 39
Chs2	DMSO	22	4778	1239	–	0	–	838	263	–	-4.5, -3, -1.5	-581	185	–	3, 4.5, 6
	LatA	22	3511	1188	73%	15	15	311	196	37%	-3, -1.5, 0	-61	53	10%	21, 22.5, 24, 25.5, 27
Chs3	DMSO	22	5490	1299	–	3	–	537	225	–	-3, -1.5, 0, 1.5	-366	208	–	6, 7.5, 9
	LatA	19	3557	1442	65%	22.5	19.5	153	118	29%	-1.5, 0, 1.5, 3	-17	44	5%	28.5, 30, 31.5, 33
Fks1	DMSO	19	26552	4995	–	4.5	–	3370	865	–	-3, -1.5, 0	-838	317	–	9, 10.5, 12, 13.5
	LatA	18	6920	2549	26%	-19.5	-24	-57	84	-10%	-3, -1.5, 0	-9	53	1%	9, 10.5, 12, 13.5

Table S4. Pearson correlation coefficient between Inn1-GFP and cytokinetic proteins. Related to Figure 6.

Rank	Protein	*r*	*P* value
1	Chs2-GFP	0.95	2.0×10^{-22}
2	GFP-Tpm1	0.93	6.8×10^{-19}
3	Mob2-3GFP	0.86	1.9×10^{-13}
4	Cdc14-GFP	0.83	1.5×10^{-11}
5	GFP-Chs4	0.74	2.6×10^{-8}
6	Mob1-mNG	0.74	2.6×10^{-8}
7	Chs3-GFP	0.66	2.3×10^{-6}
8	Bni1-GFP	0.62	1.5×10^{-5}
9	Ace2-3GFP	0.58	7.2×10^{-5}
10	GFP-Mlc1	0.50	7.8×10^{-4}
11	Cyk3-GFP	0.48	1.3×10^{-3}
12	GFP-Fks1	0.47	1.9×10^{-3}
13	Hof1-GFP	0.38	1.5×10^{-2}
14	Iqg1-GFP	0.27	8.7×10^{-2}
15	Myo2-GFP	0.25	1.1×10^{-1}
16	Abp1-3GFP	0.23	1.4×10^{-1}
17	Exo84-GFP	0.21	1.8×10^{-1}
18	Myo1-GFP	0.09	5.9×10^{-1}
19	Cdc3-GFP	-0.18	2.5×10^{-1}
20	Crh1-GFP	-0.31	5.0×10^{-2}
21	GFP-Kre6	-0.33	3.7×10^{-2}
22	Bnr1-GFP	-0.54	2.3×10^{-4}
23	Cts1-GFP	-0.60	3.4×10^{-5}

Table S5. Pearson correlation coefficient between GFP-Kre6 and cytokinetic proteins. Related to Figure 6.

Rank	Protein	*r*	*P* value
1	Crh1-GFP	0.96	8.9×10^{-25}
2	Abp1-3GFP	0.79	4.1×10^{-10}
3	Exo84-GFP	0.76	7.3×10^{-9}
4	Myo2-GFP	0.70	7.3×10^{-7}
5	GFP-Fks1	0.60	3.7×10^{-5}
6	Cyk3-GFP	0.50	8.7×10^{-4}
7	Bni1-GFP	0.33	3.6×10^{-2}
8	Chs3-GFP	0.27	9.2×10^{-2}
9	Ace2-3GFP	0.25	1.1×10^{-1}
10	Mob2-3GFP	-0.17	2.8×10^{-1}
11	GFP-Tpm1	-0.23	1.4×10^{-1}
12	Chs2-GFP	-0.25	1.1×10^{-1}
13	Cts1-GFP	-0.26	9.8×10^{-2}
14	Inn1-GFP	-0.33	3.7×10^{-2}
15	GFP-Chs4	-0.34	3.2×10^{-2}
16	Mob1-mNG	-0.34	3.2×10^{-2}
17	Hof1-GFP	-0.60	2.7×10^{-5}
18	Bnr1-GFP	-0.65	3.3×10^{-6}
19	Cdc14-GFP	-0.68	9.5×10^{-7}
20	GFP-Mlc1	-0.83	1.1×10^{-11}
21	Cdc3-GFP	-0.85	8.9×10^{-13}
22	Myo1-GFP	-0.93	2.2×10^{-19}
23	Iqg1-GFP	-0.94	9.1×10^{-20}

TRANSPARENT METHODS

Yeast media and culture conditions

Standard culture media and genetic techniques were used (Guthrie and Fink, 1991). Yeast strains were grown routinely at 25°C in synthetic complete (SC) minimal medium lacking specific amino acid(s) and/or uracil or in rich medium YM-1 (Lillie and Pringle, 1980) or yeast extract/peptone/dextrose (YPD). Neutralized SC medium (pH was adjusted to 7.0 by supplement of sodium hydroxide) was used for live-cell imaging of GFP molecule exposed to the extracellular environment (e.g., Cts1-GFP). Magic medium (MM, SC-Leu-His-Arg+Canavanine+G418) (Pan, et al., 2004) was used for *MAT*a strain selection during *kre6-ts* strain construction. Low-pH YPD medium (pH was adjusted to 4.5 by supplement of 110 mM K_2HPO_4 and 73.4 mM of citric acid, final concentrations) was used for the K1 killer toxin sensitivity test. Stock solutions of 20 mM LatA (in DMSO, FUJIFILM Wako Pure Chemical, Osaka, Japan), and 1% (w/v) CW (in distilled water, Sigma, St. Louis, MO) were diluted into media at the indicated final concentrations.

Constructions of strains

Yeast strains used in this study are listed in Table S1. Except for *kre6*-ts mutants, new strains were constructed either by integrating a plasmid carrying a modified gene at a genomic locus or by transferring a deletion or tagged allele of a gene from a plasmid or from one strain to another via PCR amplification and yeast transformation (Slubowski, et al., 2015; Lee, et al., 2013; Longtine, et al., 1998) (see footnotes in **Table S1**). For the construction of *kre6*-ts mutants, see the section below.

Primers and plasmids

All PCR primers and plasmids used in this study are listed in **Table S2**. All PCR primers were purchased from Integrated DNA Technologies (Coralville, IA). All new constructs were validated by sequencing performed at the DNA Sequencing Facility, University of Pennsylvania. Plasmids YIp128-CDC3-GFP (Gao, et al., 2007), pFA6a-link-yoEGFP-SpHIS5, pFA6a-link-yomApple-CaURA3, and pFA6a-link-yomApple-Kan (Lee, et al., 2013), pFA6a-link-GFPEnvy-SpHis5 (Slubowski, et al., 2015), pFA6a-link-yoEGFP-NatMX6 (Marquardt, et al., 2020), pFA6a-GFP(S65T)-TRP1, pFA6a-GFP(S65T)-kanMX6, pFA6a-His3MX6 and pFA6a-TRP1 (Longtine, et al., 1998), YIplac128-GFP-MLC1 (Feng, et al., 2015), and pRS305-CHS2-GFP (Wloka, et al., 2011) were described previously.

Plasmids pG366 (EXO84-GFP, integrative, *TRP1*) (Zhang, et al., 2005), bWL715 (Markus, et al., 2015), pFA6a-3GFP-KanMX6 (Wu, et al., 2006), pFA6a-URA3-KanMX6 (Onishi, et al., 2013), pJL68 (Larson, et al., 2008), pBK42 (Korinek, et al., 2000), and p4339 (Tong, et al., 2001) were generous gifts from Wei Guo (University of Pennsylvania), Wei-Lih Lee (Dartmouth College), Jian-Qiu Wu (The Ohio State University), John Pringle (Stanford University), Kelly Tatchell (Louisiana State University Health Sciences Center), John Chant (Harvard University), and Charles Boone (University of Toronto), respectively. The following plasmids were generated for this study: To generate pFA6a-GFPEnvy-KanMX6 and pFA6a-link-GFPEnvy-KanMX6, ~0.7-kb PacI-AscI fragment containing GFP^{Envy} from pFA6a-link-GFPEnvy-SpHis5 (Slubowski, et al., 2015) was subcloned to replace ~0.7-kb PacI-AscI region of either pFA6a-yEGFP-KanMX (lab stock) or pFA6a-link-yoEGFP-Kan (Addgene #44900), respectively. To generate pFA6a-link-ymNeonGreen-KanMX6, ~0.7-kb PacI-AscI fragment containing *ymNeonGreen* from pFA6a-ymNeonGreen-KanMX6, a generous gift from Takashi Ito, was subcloned to replace ~0.7-kb PacI-AscI region of pFA6a-link-yoEGFP-Kan. Plasmids pRS305-CHS2(I750A)-GFP, pRS305-CHS2(S751A)-GFP, and pRS305-CHS2(N797Q)-GFP were constructed by PCR-mediated site-directed mutagenesis (Carey, et al., 2013) using the plasmid pRS305-CHS2-GFP as the template DNA and the pair of primers P305 and P595, P306 and P596, and P307 and P597, respectively. Of note, during sequence validation, we found all plasmids, including the

template plasmid, carry unintended mutation corresponding to the deletion of the last five residues of Chs2 (aa 959–963). The following experiments confirmed that the deletion did not lead to a significant change in Chs2 kinetics at the division site (e.g., Figure 2C vs. Figure 5C). Plasmids pRS315-KRE6 and pRS315-ENVY-KRE6 were constructed by recombination-mediated plasmid construction (Oldenburg, et al., 1997). A DNA fragment containing *KRE6* or *GFP^{Envy}-KRE6,* ORF flanked by ~1-kb promoter and ~0.5-kb terminator regions were amplified by PCR from the chromosomal DNA of YEF8552 (lab stock, YEF473A background carrying WT *KRE6* gene) or YEF8378 (*GFP^{Envy}-KRE6*, see **Table S1** for details) and the pair of primers P229 and P606. Resultant PCR products were then assembled with XbaI-, NotI-, and BamHI-linearized pRS315 in yeast cells. Plasmids pRS305-KRE6^{QNQ} and pRS315-ENVY-KRE6^{QNQ} were generated by PCR-mediated site-directed mutagenesis using the plasmid pRS305-KRE6 or pRS315-ENVY-KRE6, respectively, as the template DNA and the pair of primers P317 and P605. To generate pRS305-KRE6 and pRS305-ENVY-KRE6^{QNQ}, ~5.3-kb or ~6.0-kb BglI fragment containing *KRE6* or *GFP^{Envy}-kre6^{QNQ}* from either pRS315-KRE6 or pRS315-ENVY-KRE6^{QNQ}, respectively, was ligated into the 3.8-kb BglI backbone from pRS305. To generate pRS305-ENVY-KRE6(1-341), a DNA fragment carrying partial ORF of *GFP^{Envy}-KRE6* (from ~1-kb upstream of the start codon to the residue 341 of *KRE6* followed by a new stop codon) was amplified by PCR using the chromosomal DNA of YEF8378 as the template DNA and the pair of primers P229 and P522. The resultant PCR product was then subcloned into ApaI- and SacI-digested pRS305 using the Quick-Fusion cloning kit (Bimake, Houston, TX, USA). To generate pRS316-ENVY-FKS1(1-789), a DNA fragment carrying partial ORF of *GFP^{Envy}-FKS1* (from ~1-kb upstream of the start codon to the residue 789 of *FKS1* followed by a new stop codon) was amplified by PCR using the chromosomal DNA of YEF8407 (*GFP^{Envy}-FKS1*, see **Table S1** for details) as the template DNA and the pair of primers P222 and P512. The resultant PCR product was then assembled with ApaI- and SacI-linearized pRS316 in yeast cells by recombination-mediated plasmid construction. The same *GFP^{Envy}-fks1(1-789)* fragment was amplified by PCR using the pRS316-ENVY-FKS1(1-789) as the template DNA and the pair of primers

P222 and P512. The resultant PCR product was then subcloned into ApaI- and SacI-digested pRS307 (Addgene #51785) using the Quick-Fusion cloning kit to generate pRS307-ENVY-FKS1(1-789). To generate pRS316-KRE6 (pYO3165), a ~4.0-kb SalI-BamHI fragment from pYO3162 [lab stock, pBluescript KS (+) backbone, carrying ~4.0-kb ClaI-ApaI region containing yeast chromosomal *KRE6* subcloned from YGPM10h21 (Yeast Genomic Tiling Collection, GE Healthcare, Chicago, IL, USA), sandwiched by SalI-BamHI site] was subcloned into SalI-BamHI site of pRS316 (low-copy, *URA3*). To generate YIp128-proHIS3-yEGFP-TPM1-tADH1, a DNA fragment containing *TPM1* ORF was amplified by PCR using YIp128-proACT1-TPM1-yeGFP-tADH1 (lab stock, carrying WT *TPM1*) as the template DNA and set of primers P288 and P580 with the introduction of SpeI site followed by GGSGGTGG linker and AscI-site, at 5' and 3' ends of *TPM1* ORF, respectively. The resultant PCR product was digested by SpeI and AscI and then was ligated into SpeI- and AscI-digested YIp128-proHIS3-GFP-ECM25-(536-588AA)-tADH1 [lab stock, integrative, *LEU2*, expresses N-terminally GFP-tagged Ecm25 (aa 536–588) by *HIS3* promoter] to replace the insert of ECM25-(536-588AA). The resultant plasmid carries the expression cassette of N-terminally GFP-tagged Tpm1 under *HIS3* promoter control. Plasmid YEplac112-CYK3$^{TGc\Delta}$ was constructed by inverse PCR-mediated fusion cloning (Raman and Martin, 2014). A DNA fragment containing the entire template plasmid except the TGc region of *CYK3* (aa 516–581) was amplified by inverse PCR using pBK42 (YEplac112-CYK3) (Korinek, et al., 2000) as the template DNA and primers with P632 and P633. The resultant PCR product was self-assembled by the Quick-Fusion cloning kit.

Generation of *kre6*-ts mutants

Temperature-sensitive mutations in *KRE6* (i.e. *kre6-ts* mutations) were made by plasmid-based diploid shuffle (Ben-Aroya, et al., 2010). In brief, error-prone PCR (Cadwell and Joyce, 1992) was performed using pYO3165 as a template DNA to generate a library of mutagenized *KRE6* fragments, hereafter *kre6**, which contained the full-length *kre6** ORF with ~0.5-kb flanking regions of promoter and terminator. The resultant PCR product and

HindIII-linearized vector pYO3166 (lab stock, identical to pYO3165 except for ~1.7-kb HindIII-SacI region, which includes the ~1.5-kb *KRE6* ORF, was replaced by a short linker) were mixed and then assembled into plasmids by a recombination-based method in YOC4875 (lab stock, *KRE6*/*kre6*Δ::*kanMX4 skn1*Δ/*skn1*Δ *CAN1*/*can1*Δ::*LEU2-MFA1pr-HIS3*). Ura+ transformants were sporulated, plated on MM-Ura, and then incubated at 25°C for 3 days to select *MAT*a haploid strains carrying a plasmid-borne *kre6** allele (*MAT*a *kre6*Δ::*kanMX4 skn1*Δ *can1*Δ::*LEU2-MFA1pr-HIS3* [CEN, *URA3*, *kre6**]). Obtained colonies were replica-plated to new MM-Ura plates and incubated at either the permissive (25°C) or restrictive (37°C) temperature for two days. Cells that grew under 25°C but not under 37°C were selected as *kre6-ts* candidates to purify the plasmid. The *kre6-ts* mutations were verified by sequencing.

To validate these mutations as bona fide *kre6-ts* alleles, ~4.0-kb SalI-BamHI fragments from plasmids were subcloned into SalI-BamHI site of SB221, a *KanMX4–URA3* switcher cassette plasmid and a generous gift from Philip Hieter (Ben-Aroya, et al., 2008). The resultant plasmids were digested by NotI within the switcher cassette fragment, generating a linearized fragment harboring either $kre6^{N461S}$- or $kre6^{E582G}$-*URA3* that was sandwiched by the 5' and 3' fragments of *KanMX4*. The linearized fragments were used for transformation of YOC4875 to replace chromosomal *kre6*Δ::*KanMX* by *kre6-ts-URA3* cassette. Ura+ transformants were sporulated, plated on MM-Ura-G418, and then incubated at 25°C for 3 days to select *MAT*a haploid strain carrying *kre6-ts* allele at its endogenous locus (*MAT*a *kre6*Δ::*kanmx4::kre6-ts-URA3 skn1*Δ *can1*Δ::*LEU2-MFA1pr-HIS3*). After confirming their ts phenotype, these strains were crossed with *MAT*α *skn1*Δ::*KanMX4* (EUROSCARF) and then subjected to tetrad dissection to eliminate the *MAT*a selection marker as well as to confirm the 2:2 Mendelian segregation patterns for the ts mutations.

Imaging and data analysis

For time-lapse microscopy, cells were cultured to exponential phase at 25°C in SC medium, briefly sonicated at 15% power for 5 seconds to declump (model Q55, Qsonica, Newtown, CT, USA), concentrated by centrifugation, and spotted onto a poly-lysine-coated glass-bottom dish, and then embedded with SC containing agarose (Okada, et al., 2017). For imaging experiments with drug perfusion, cells were cultured and harvested as aforementioned and spotted onto concanavalin A (Sigma)-coated glass-bottom dish, and then drug-containing SC medium was added. Images were acquired at room temperature (23°C) by a spinning-disk confocal microscope (model Eclipse Ti-U, Nikon, Tokyo, Japan) with a 100x/1.49NA oil objective (model CFI Apo TIRF 100x, Nikon), combined with a confocal scanner unit (model CSU-X1, Yokogawa, Tokyo, Japan). An EMCCD camera (model Evolve 512 Delta, Photometrics, Tucson, AZ, USA) was used for capture. Solid-state lasers for excitation (405 nm for CW, 488 nm for GFP and 561 nm for RFP) were housed in a launch (model ILE-400, Spectral Applied Research, Richmond Hill, ON, Canada). The imaging system was controlled by MetaMorph version 7.8.10.0 (Molecular Devices, San Jose, CA, USA). Images were taken every 1 or 1.5 min with 12 z-stacks with a step-size of 0.7 μm. A sum or max projection was created with NIH ImageJ (1.51h). For the quantification of fluorescence intensities, the integrated density at the division site from sum projected images was calculated by subtracting the fluorescence intensity in the background area from the total intensity in an ImageJ-drawn polygon covering the division site. For calculating of constriction rate, we manually measured the myosin ring diameter during constriction from max projection images acquired from time-lapse imaging, and then calculate the slope of the diameter curve from 4 or 5-time points including midpoint of constriction. Data analyses were performed with Microsoft Excel and R (ver. 3.0.1).

Quantification of chitin deposition in PS

To determine the relative chitin levels at the division site (data presented in **Figure 5E**), cells were grown to exponential phase in liquid SC-Trp at 25°C. Cells were pelleted by

centrifugation and washed once with SC with 10 μg/ml CW (Sigma). Cells were then resuspended in the same medium and subjected to the imaging.

For the quantification of CW intensities, the integrated density at the division site from sum projected images was measured by subtracting the background intensity from the total intensity of the region of interest covered by ImageJ-drawn polygon. Cells at the later stage of cytokinesis, judged by breakage of mitotic spindle and completion of myosin constriction, were used for analysis.

Yeast growth assay

Spot assay was performed to examine ts phenotype and sensitivity to CW of cells. Cells were cultured in YPD medium at 25°C for 12 hours, and cell culture was diluted with fresh YPD medium to the 0.3 OD_{600}. The cell suspension was subjected to 10-fold serial dilutions and inoculated as 5 μl of spot onto YPD-, or YPD-plate containing CW. After incubation at 25°C or 37°C for 3–6 days, the cell growth on the plate was recorded. The growth curve of cells in liquid media was measured to determine ts phenotype. Cells were cultured to exponential phase at 25°C in YPD medium and then split into two and diluted with fresh YPD medium to the 0.1 OD_{600}. Split cultures were incubated in a water bath shaker set at 25°C or 37°C for 10 hours, and OD_{600} values were measured every 2 hours for 25°C samples and 1.5 hours for 37°C samples.

K1 killer toxin sensitivity assay

Cells were cultured to exponential phase at 25°C in YM-1 medium and diluted with fresh low-pH YPD medium to the 0.25 OD_{600}. The culture medium was mixed with a pre-warmed low-pH YPD medium containing low-melting agarose (final 1%, SeaPlaque Agarose, Lonza, Basel, Switzerland) and poured onto a low-pH YPD plate to make a yeast lawn. Then, spot 5 μl of an overnight culture (in YM-1 and at 25°C) of the K1 killer toxin-

producing strain (NCYC232, at 1.4 OD_{600}). After incubation at 25°C for three days, the cell growth on the plate was recorded. ImageJ was used to measure the width of the growth inhibition zone.

Quantification and statistical analysis

For the statistical analyses on chitin levels at the division site (related to **Figure 5E**) and K1 killer toxin sensitivity (related to **Figure 6F**), a two-sided unpaired t-test (assuming unequal variances) was performed. For the correlation analysis on kinetic signature (related to **Tables S4** and **S5**), a Pearson correlation coefficient between mean values of protein accumulation kinetics from selected time points (-10 to +30 min) was calculated. "n" refers to the number of cells analyzed unless indicated otherwise.

SUPPLEMENTAL REFERENCES

Ben-Aroya, S., Coombes, C., Kwok, T., O'Donnell, K.A., Boeke, J.D., and Hieter, P. (2008). Toward a comprehensive temperature-sensitive mutant repository of the essential genes of *Saccharomyces cerevisiae*. Mol. Cell *30*, 248-258.
Ben-Aroya, S., Pan, X., Boeke, J.D., and Hieter, P. (2010). Making temperature-sensitive mutants. Methods Enzymol. *470*, 181-204.
Bi, E., and Pringle, J.R. (1996). *ZDS1* and *ZDS2*, genes whose products may regulate Cdc42p in *Saccharomyces cerevisiae*. Mol. Cell. Biol. *16*, 5264-5275.
Brachmann, C.B., Davies, A., Cost, G.J., Caputo, E., Li, J., Hieter, P., and Boeke, J.D. (1998). Designer deletion strains derived from *Saccharomyces cerevisiae* S288C: a useful set of strains and plasmids for PCR-mediated gene disruption and other applications. Yeast *14*, 115-132.
Cadwell, R.C., and Joyce, G.F. (1992). Randomization of genes by PCR mutagenesis. PCR methods Applic. *2*, 28-33.
Carey, M.F., Peterson, C.L., and Smale, S.T. (2013). PCR-mediated site-directed mutagenesis. Cold Spring Harb. Protoc. *2013*, 738-742.
Eriksson, P., Thomas, L.R., Thorburn, A., and Stillman, D.J. (2004). pRS yeast vectors with a LYS2 marker. Biotechniques *36*, 212-213.
Feng, Z., Okada, S., Cai, G., Zhou, B., and Bi, E. (2015). MyosinII heavy chain and formin mediate the targeting of myosin essential light chain to the division site before and during cytokinesis. Mol. Biol. Cell *26*, 1211-1224.
Gao, X.D., Sperber, L.M., Kane, S.A., Tong, Z., Hin Yan Tong, A., Boone, C., and Bi, E. (2007). Sequential and distinct roles of the cadherin domain-containing protein Axl2p in cell polarization in yeast cell cycle. Mol. Biol. Cell *18*, 2542-2560.
Gietz, R.D., and Sugino, A. (1988). New yeast-*Escherichia coli* shuttle vectors constructed with in vitro mutagenized yeast genes lacking six-base pair restriction sites. Gene *74*, 527-534.
Guthrie, C., and Fink, G.R. (1991). Guide to Yeast Genetics and Molecular Biology. Methods Enzymol. Vol. 194, 933 pp.
Korinek, W.S., Bi, E., Epp, J.A., Wang, L., Ho, J., and Chant, J. (2000). Cyk3, a novel SH3-domain protein, affects cytokinesis in yeast. Curr. Biol. *10*, 947-950.
Kurita, T., Noda, Y., Takagi, T., Osumi, M., and Yoda, K. (2011). Kre6 protein essential for yeast cell wall beta-1,6-glucan synthesis accumulates at sites of polarized growth. J. Biol. Chem. *28*6, 7429-7438.
Larson, J.R., Bharucha, J.P., Ceaser, S., Salamon, J., Richardson, C.J., Rivera, S.M., and Tatchell, K. (2008). Protein phosphatase type 1 directs chitin synthesis at the bud neck in *Saccharomyces cerevisiae*. Mol. Biol. Cell *19*, 3040-3051.
Lee, S., Lim, W.A., and Thorn, K.S. (2013). Improved blue, green, and red fluorescent protein tagging vectors for *S. cerevisiae*. PLoS One *8*, e67902.
Lillie, S.H., and Pringle, J.R. (1980). Reserve carbohydrate metabolism in *Saccharomyces cerevisiae*: responses to nutrient limitation. J. Bacteriol. *143*, 1384-1394.
Longtine, M.S., McKenzie, A., III,, DeMarini, D.J., Shah, N.G., Wach, A., Brachat, A., Philippsen, P., and Pringle, J.R. (1998). Additional modules for versatile and economical

PCR-based gene deletion and modification in *Saccharomyces cerevisiae*. Yeast *14*, 953-961.
Markus, S.M., Omer, S., Baranowski, K., and Lee, W.L. (2015). Improved Plasmids for Fluorescent Protein Tagging of Microtubules in *Saccharomyces cerevisiae*. Traffic 16, 773-786.
Marquardt, J., Yao, L.L., Okada, H., Svitkina, T., and Bi, E. (2020). The LKB1-like kinase Elm1 controls septin hourglass assembly and stability by regulating filament pairing. Curr. Biol. *30*, 2386-2394 e4.
Oh, Y., Chang, K.J., Orlean, P., Wloka, C., Deshaies, R., and Bi, E. (2012). Mitotic exit kinase Dbf2 directly phosphorylates chitin synthase Chs2 to regulate cytokinesis in budding yeast. Mol. Biol. Cell *23*, 2445-2456.
Okada, S., Wloka, C., and Bi, E. (2017). Analysis of protein dynamics during cytokinesis in budding yeast. Methods Cell Biol. *137*, 25-45.
Oldenburg, K.R., Vo, K.T., Michaelis, S., and Paddon, C. (1997). Recombination-mediated PCR-directed plasmid construction in vivo in yeast. Nucleic Acids Res. *25*, 451-452.
Onishi, M., Ko, N., Nishihama, R., and Pringle, J.R. (2013). Distinct roles of Rho1, Cdc42, and Cyk3 in septum formation and abscission during yeast cytokinesis. J. Cell Biol. *202*, 311-329.
Pan, X., Yuan, D.S., Xiang, D., Wang, X., Sookhai-Mahadeo, S., Bader, J.S., Hieter, P., Spencer, F., and Boeke, J.D. (2004). A robust toolkit for functional profiling of the yeast genome. Mol. Cell *16*, 487-496.
Raman, M., and Martin, K. (2014). One solution for cloning and mutagenesis: In-fusion HD cloning plus. Nat. Methods *11*, iii-v.
Sikorski, R.S., and Hieter, P. (1989). A system of shuttle vectors and yeast host strains designed for efficient manipulation of DNA in *Saccharomyces cerevisiae*. Genetics *122*, 19-27.
Slubowski, C.J., Funk, A.D., Roesner, J.M., Paulissen, S.M., and Huang, L.S. (2015). Plasmids for C-terminal tagging in *Saccharomyces cerevisiae* that contain improved GFP proteins, Envy and Ivy. Yeast *32*, 379-387.
Tong, A.H., Evangelista, M., Parsons, A.B., Xu, H., Bader, G.D., Page, N., Robinson, M., Raghibizadeh, S., Hogue, C.W., Bussey, H., et al. (2001). Systematic genetic analysis with ordered arrays of yeast deletion mutants. Science *294*, 2364-2368.
Wloka, C., Nishihama, R., Onishi, M., Oh, Y., Hanna, J., Pringle, J.R., Krauss, M., and Bi, E. (2011). Evidence that a septin diffusion barrier is dispensable for cytokinesis in budding yeast. Biol. Chem. *392*, 813-829.
Wloka, C., Vallen, E.A., Thé, L., Fang, X., Oh, Y., and Bi, E. (2013). Immobile myosin-II plays a scaffolding role during cytokinesis in budding yeast. J. Cell Biol. *200*, 271-286.
Wu, J.Q., Sirotkin, V., Kovar, D.R., Lord, M., Beltzner, C.C., Kuhn, J.R., and Pollard, T.D. (2006). Assembly of the cytokinetic contractile ring from a broad band of nodes in fission yeast. J. Cell Biol. *174*, 391-402.
Yabe, T., Yamada-Okabe, T., Nakajima, T., Sudoh, M., Arisawa, M., and Yamada-Okabe, H. (1998). Mutational analysis of chitin synthase 2 of *Saccharomyces cerevisiae*. Identification of additional amino acid residues involved in its catalytic activity. Eur. J. Biochem. 258, 941-947.

Zhang, X., Zajac, A., Zhang, J., Wang, P., Li, M., Murray, J., TerBush, D., and Guo, W. (2005). The critical role of Exo84p in the organization and polarized localization of the exocyst complex. J. Biol. Chem. *280*, 20356-20364.
